# Supplementary material for: Ultrasound stimulation of the motor cortex during tonic muscle contraction
Source: PLoS One. 2022 Apr 20;17(4):e0267268. doi: 10.1371/journal.pone.0267268 (PMC9020726; doi:10.1371/journal.pone.0267268)
Supplement: S1 Fig — Top) Sliding window standard deviation trace of a single trial EMG trace. The black horizontal line marks the detection threshold. The vertical green lines mark the beginning and end of the detected cSP. Bottom) The original high-pass filtered EMG trace. (PDF) [file pone.0267268.s001.pdf]

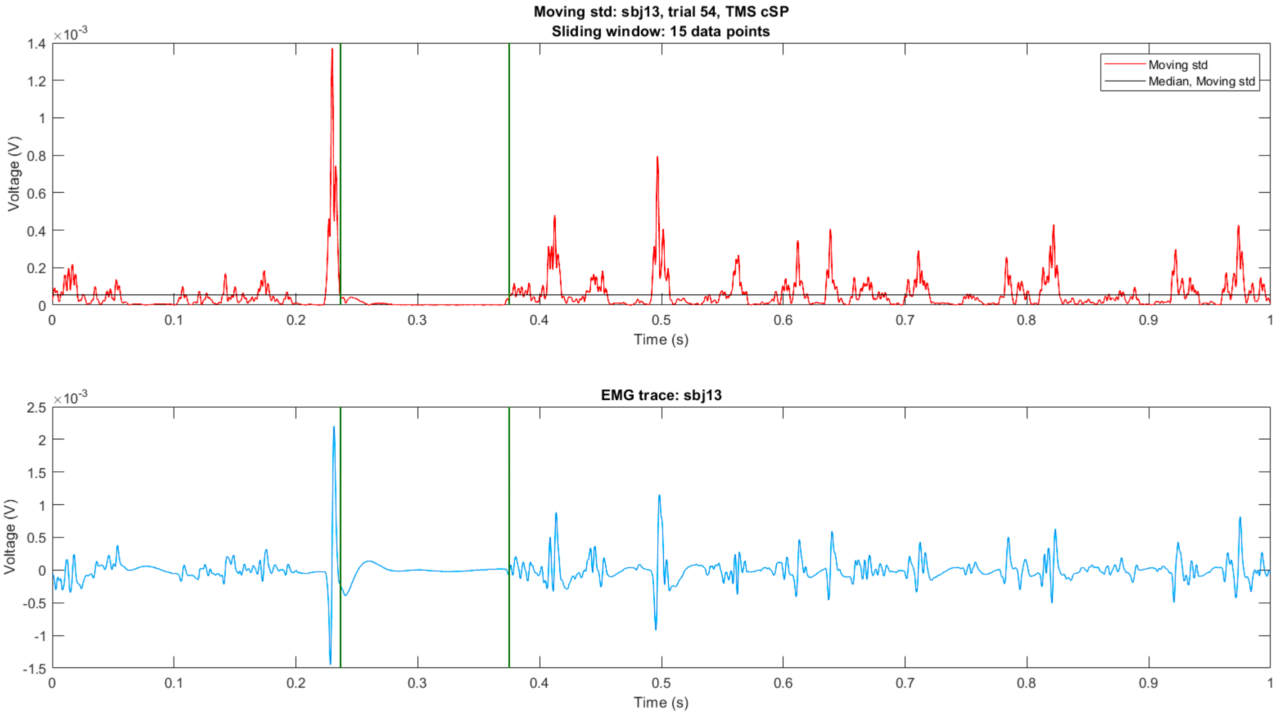

S1 Fig. Visualization of the automated cSP detection method. *Top) Sliding window standard deviation trace of a single trial EMG trace. The black horizontal line marks the detection threshold. The vertical green lines mark the beginning and end of the detected cSP. Bottom) The original high-pass filtered EMG trace.*

Supporting information for:

Ultrasound stimulation of the motor cortex during tonic muscle contraction
Ian S. Heimbuch, Tiffany K. Fan, Allan Wu, Guido C. Faas, Andrew C. Charles, Marco Iacoboni
