# Supplementary material for: Ultrasound stimulation of the motor cortex during tonic muscle contraction
Source: PLoS One. 2022 Apr 20;17(4):e0267268. doi: 10.1371/journal.pone.0267268 (PMC9020726; doi:10.1371/journal.pone.0267268)
Supplement: S2 Fig — Level of EMG activity during different sections of tUS trials. Left) Baseline, -200 to -50 ms before onset. Right) 0 to 150 ms after tUS onset (first 150 ms of tUS exposure). Values were normalized via dividing by the trial mean. (PDF) [file pone.0267268.s002.pdf]

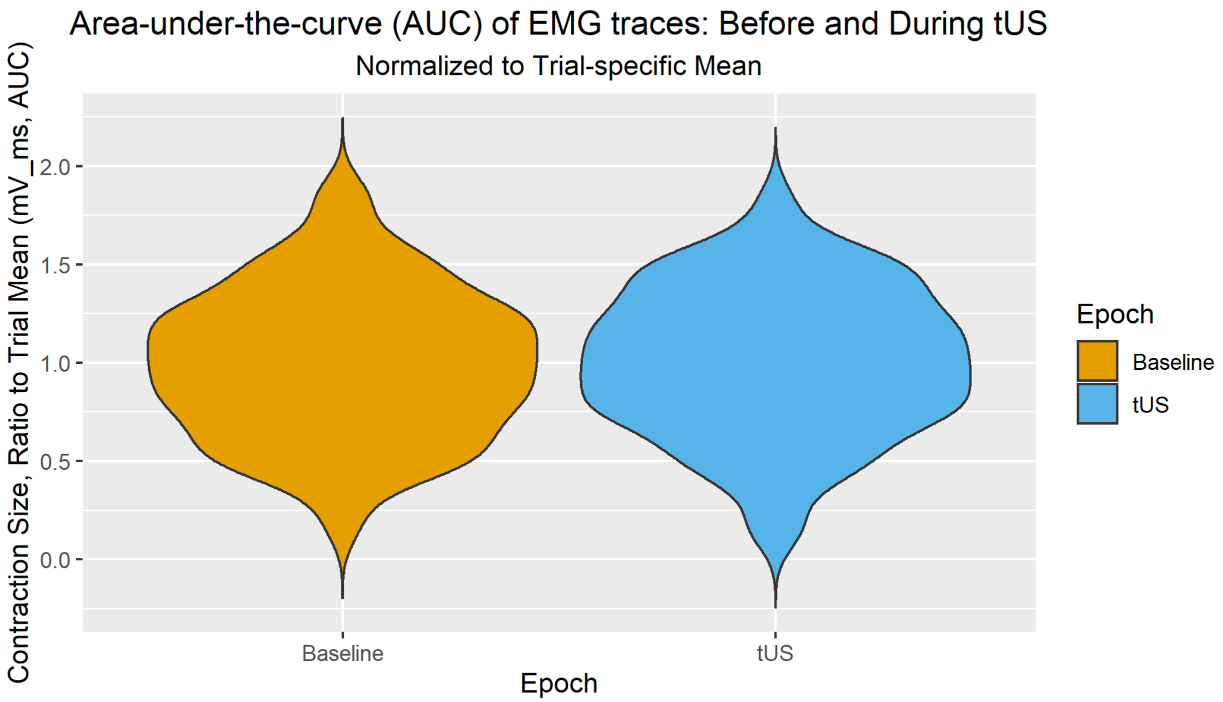

S2* Fig. *Area-under-the-curve of tUS traces. *Level of EMG activity during different sections of tUS trials. Left) Baseline, -200 to -50 ms before onset. Right) 0 to 150 ms after tUS onset (first 150 ms of tUS exposure). Values were normalized via dividing by the trial mean.*
