# Supplementary material for: Ultrasound stimulation of the motor cortex during tonic muscle contraction
Source: PLoS One. 2022 Apr 20;17(4):e0267268. doi: 10.1371/journal.pone.0267268 (PMC9020726; doi:10.1371/journal.pone.0267268)
Supplement: S3 Fig — Rate of EMG peaks during tonic contraction per subject. Left) TMS trials. The drop in EMG activity due to TMS-induced cSPs is visible soon after TMS onset at 0.2 s. Right) tUS trials. tUS on from 0.2–0.5 s A sliding window approach (1-ms steps) checked if an EMG peak occurred during a 5-ms time window following that point. Peaks were detected using the ‘findpeaks’ function in MATLAB. Each row contains data for all trials per subject. Color shows percentage of trials that had a peak during that time window. Percentage data was smoothed with a moving mean (~6-ms window). EMG traces were high pass filtered at 10 Hz. (PDF) [file pone.0267268.s003.pdf]

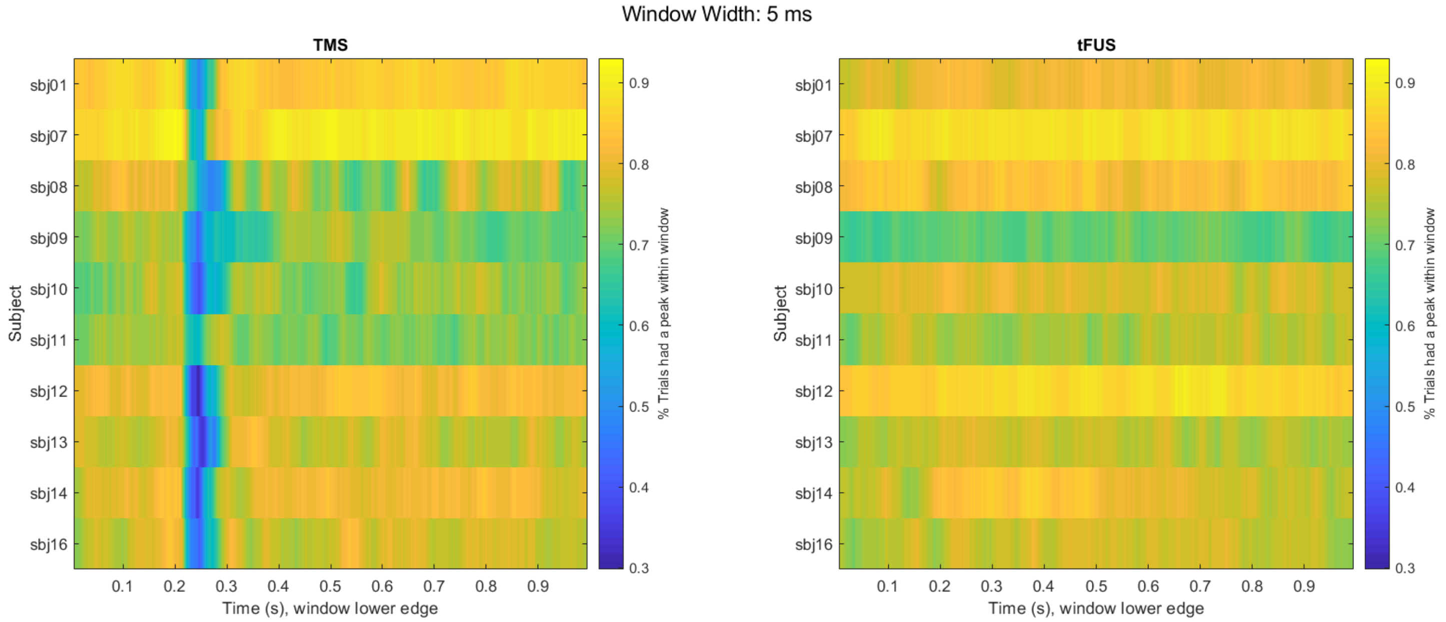

S3* Fig. *Prevalence of EMG peaks. *Rate of EMG peaks during tonic contraction per subject. Left) TMS trials. The drop in EMG activity due to TMS-induced cSPs is visible soon after TMS onset at 0.2 s. Right) tUS trials. tUS on from 0.2-0.5 s A sliding window approach (1-ms steps) checked if an EMG peak occurred during a 5-ms time window following that point. Peaks were detected using the 'findpeaks' function in MATLAB. Each row contains data for all trials per subject. Color shows percentage of trials that had a peak during that time window. Percentage data was smoothed with a moving mean (~6-ms window). Left) TMS trials. Right) tUS trials. EMG traces were high pass filtered at 10 Hz.*
