# Supplementary material for: Ultrasound stimulation of the motor cortex during tonic muscle contraction
Source: PLoS One. 2022 Apr 20;17(4):e0267268. doi: 10.1371/journal.pone.0267268 (PMC9020726; doi:10.1371/journal.pone.0267268)
Supplement: S4 Fig — One dot per trial per condition (‘Off’ and ‘On’). EMG traces were bandpass filtered to 10–800 Hz. (PDF) [file pone.0267268.s004.pdf]

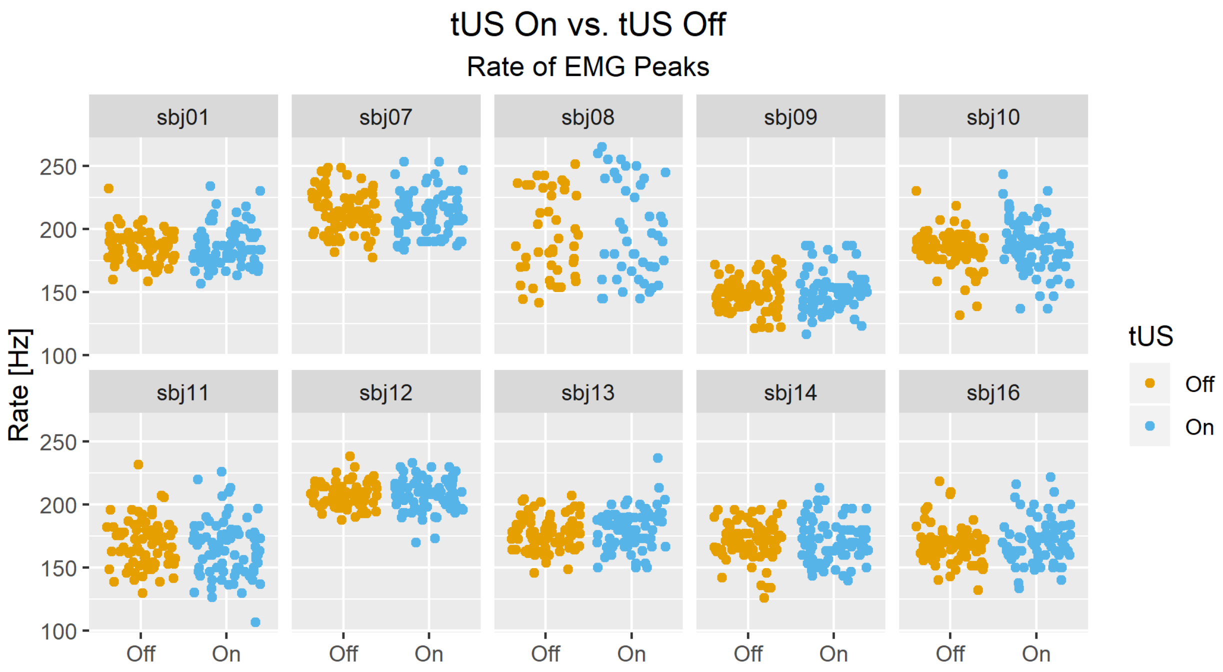

S4 Fig. Rate of EMG peaks during a single tUS trial. *One dot per trial per condition ('Off' and 'On'). EMG traces were bandpass filtered to 10-800 Hz.*

Supporting information for:

Ultrasound stimulation of the motor cortex during tonic muscle contraction
Ian S. Heimbuch, Tiffany K. Fan, Allan Wu, Guido C. Faas, Andrew C. Charles, Marco Iacoboni
