# Supplementary material for: Ultrasound stimulation of the motor cortex during tonic muscle contraction
Source: PLoS One. 2022 Apr 20;17(4):e0267268. doi: 10.1371/journal.pone.0267268 (PMC9020726; doi:10.1371/journal.pone.0267268)
Supplement: S5 Fig — (Top) Distribution of silence lengths when cSP length algorithm is run from different time points along each EMG from a contracting finger. Sliding window approach was used to bootstrap these values, with a sliding window step size of 0.001 seconds. Histogram bin width 0.001 seconds. “Null” data were the one-second tonic contraction trials during tUS exposure. tUS trials were deemed valid as null EMG traces since we saw no change in EMG traces between tUS on vs. tUS off (Figs 2 and 3, S4 Fig). The first and last 50 ms were removed to avoid boundary effects. 686,457 sliding window samples (Bottom) Lengths of silent periods for trials grouped into three categories: a clear MEP was present (“MEP”, blue), a small peak that may have been an MEP was present (“Stub”, green), and no detectable peak was present (“None”, red). Null distribution (see Top) overlayed in orange. MEP: 361. Stub: 151. None: 18. (PDF) [file pone.0267268.s005.pdf]

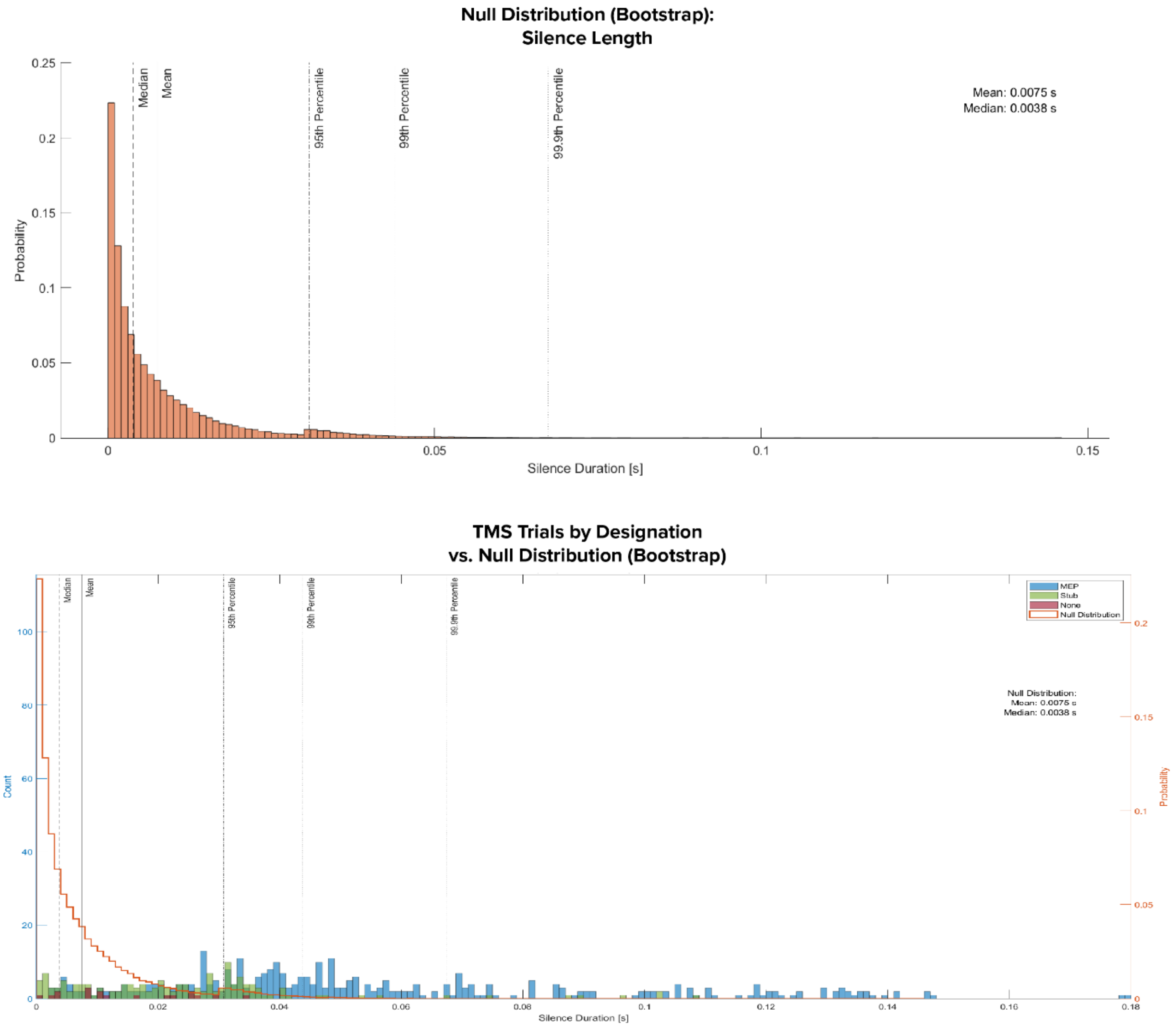


***S5 Fig. Silence null distribution bootstrap.*** *(Top) Distribution of silence lengths when cSP length algorithm is run from different time points along each EMG from a contracting finger. Sliding window approach was used to bootstrap these values, with a sliding window step size of 0.001 seconds. Histogram bin width 0.001 seconds. "Null" data were the one-second tonic contraction trials during tUS exposure. tUS trials were deemed valid as null EMG traces since we saw no change in EMG traces between tUS on vs. tUS off (Fig 3, Fig 4, S4 Fig). The first and last 50 ms were removed to avoid boundary effects. 686,457 sliding window samples (Bottom) Lengths of silent periods for trials grouped into three categories: a clear MEP was present ("MEP", blue), a small peak that may have been an MEP was present ("Stub", green), and no detectable peak was present ("None", red). Null distribution (see Top) overlayed in orange. MEP: 361. Stub: 151. None: 18.*
