# Supplementary material for: Ultrasound stimulation of the motor cortex during tonic muscle contraction
Source: PLoS One. 2022 Apr 20;17(4):e0267268. doi: 10.1371/journal.pone.0267268 (PMC9020726; doi:10.1371/journal.pone.0267268)
Supplement: S6 Fig — X-axis: Time [s]. TMS onset at 0 s. Examples are from multiple subjects. (PDF) [file pone.0267268.s006.pdf]

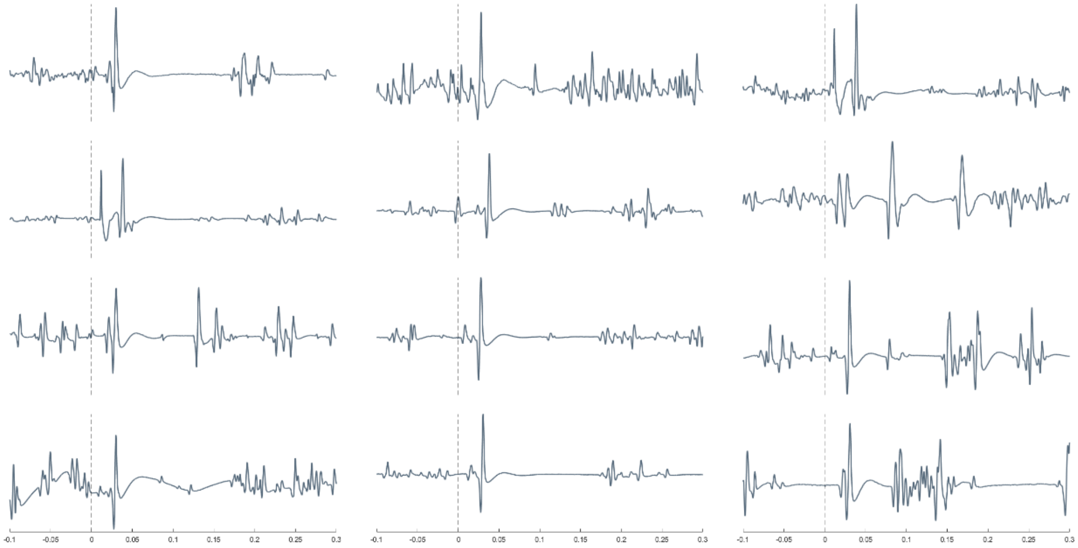

S6 Fig. Examples of cSPs with late excitatory potentials (LEPs). X*-axis: Time [s]. TMS onset at 0 s. Examples are from multiple subjects.*

Supporting information for:

Ultrasound stimulation of the motor cortex during tonic muscle contraction

Ian S. Heimbuch, Tiffany K. Fan, Allan Wu, Guido C. Faas, Andrew C. Charles, Marco Iacoboni
