# Supplementary material for: Ultrasound stimulation of the motor cortex during tonic muscle contraction
Source: PLoS One. 2022 Apr 20;17(4):e0267268. doi: 10.1371/journal.pone.0267268 (PMC9020726; doi:10.1371/journal.pone.0267268)
Supplement: S7 Fig — All subjects; all trials. EMG traces were bandpass filtered to 10–800 Hz. Same data as shown per subject in S4 Fig. Difference of the mean rates of EMG peaks were only marginally lower for tUS ‘On’ vs. tUS ‘Off’ (Delta: -0.91 Hz; 95% CI: -1.99, 0.16 Hz; p = 0.095; paired t-test). (PDF) [file pone.0267268.s007.pdf]

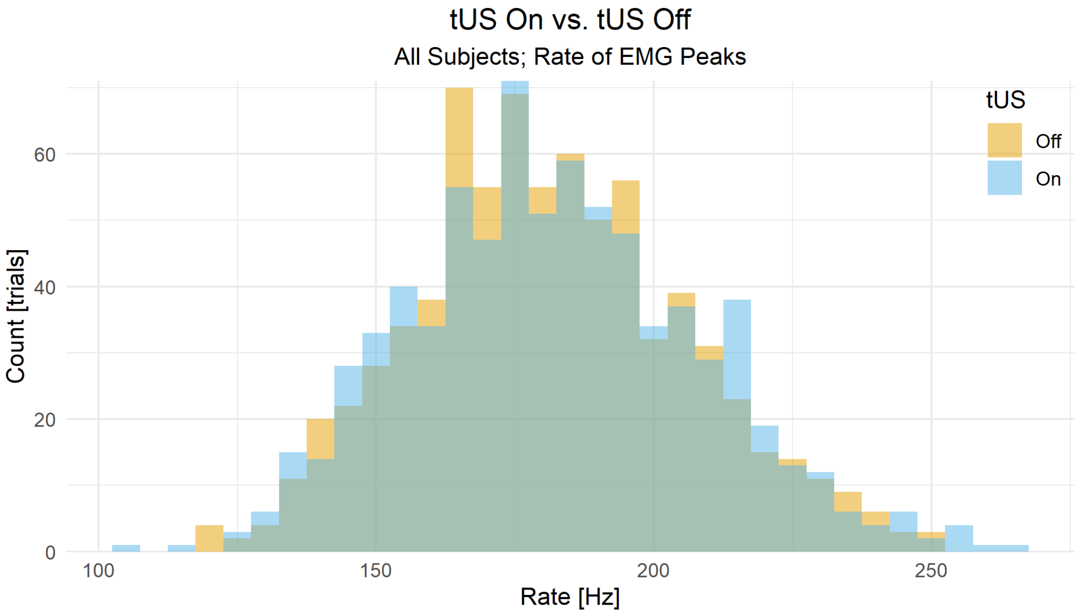


***S7 Fig. Distributions of rate of EMG peaks during a single tUS trial.*** *All subjects; all trials. EMG traces were bandpass filtered to 10-800 Hz. Same data as shown per subject in S4 Fig. Difference of the mean rates of EMG peaks were only marginally lower for tUS 'On' vs. tUS 'Off' (Delta: -0.91 Hz; 95% CI: -1.99, 0.16 Hz; p = 0.095; paired t-test).*
