# Supplementary material for: Ultrasound stimulation of the motor cortex during tonic muscle contraction
Source: PLoS One. 2022 Apr 20;17(4):e0267268. doi: 10.1371/journal.pone.0267268 (PMC9020726; doi:10.1371/journal.pone.0267268)
Supplement: S8 Fig — Same data as shown in Fig 4. Sbj08 subject was excluded from Fig 4 since their cSP trials were not used in analysis (due to use of different TMS levels). Non-zero silence durations were recorded for 90% aMT for two reasons. First, there are inherent gaps between EMG peaks during tonic contraction, which average ~7.5 ms with our algorithm and our data (S5 Fig, Top). Second, TMS of M1 results in a distribution of responses, with some trials reaching MEP and cSP threshold while other trials do not (i.e. motor thresholds are never hard cutoffs). (PDF) [file pone.0267268.s008.pdf]

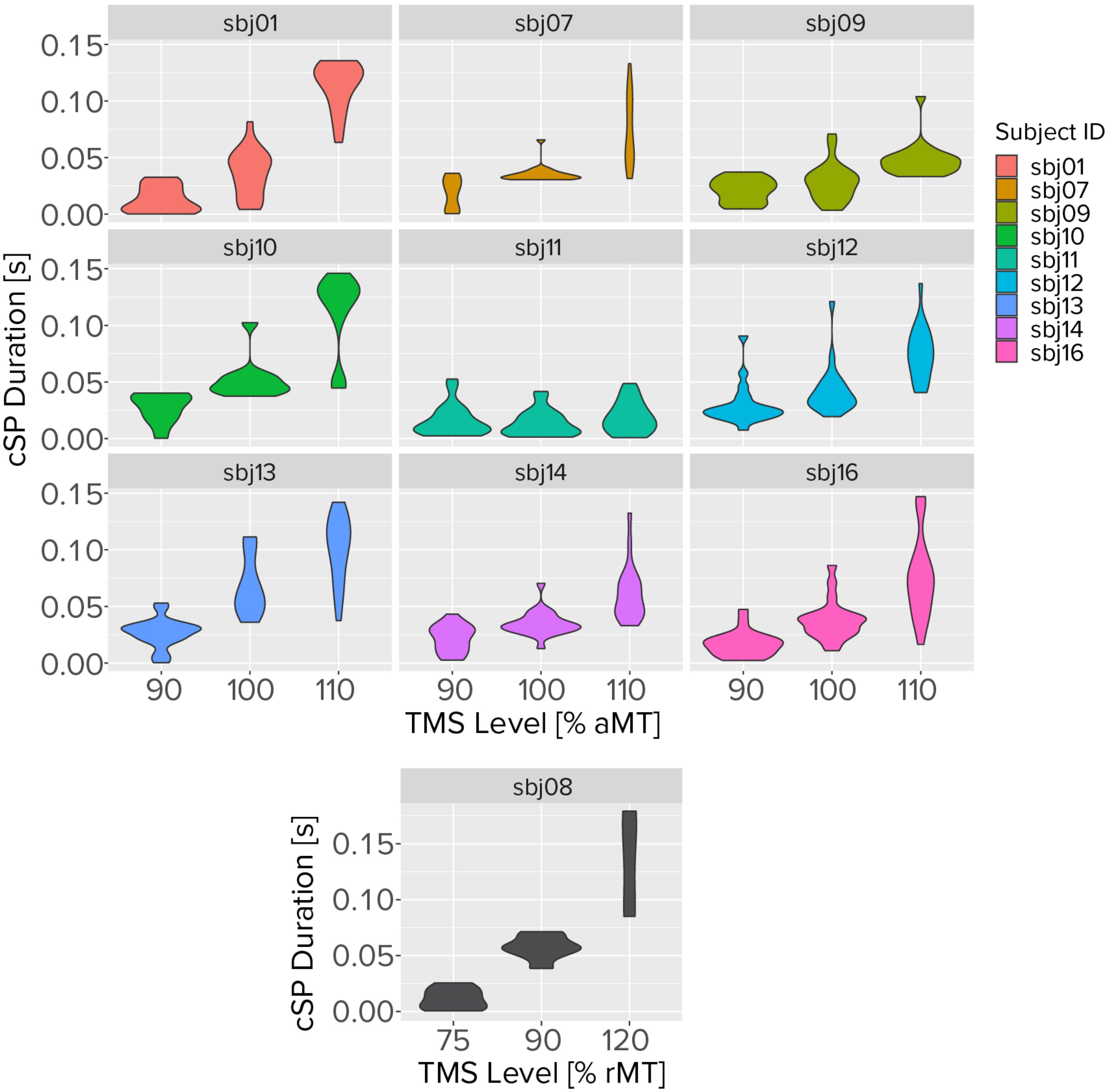

S8 Fig. Violin plots of TMS-evoked cSP durations separated by research participant *("Subject ID"). Same data as shown in Fig 5. sbj08 subject was excluded from Fig 5 since their cSP trials were not used in analysis (due to use of different TMS levels). Non-zero silence durations were recorded for 90% aMT for two reasons. First, there are inherent gaps between EMG peaks during tonic contraction, which average ~7.5 ms with our algorithm and our data (S5 Fig, Top). Second, TMS of M1 results in a distribution of responses, with some trials reaching MEP and cSP threshold while other trials do not (i.e. motor thresholds are never hard cutoffs).*

Supporting information for:

Ultrasound stimulation of the motor cortex during tonic muscle contraction
Ian S. Heimbuch, Tiffany K. Fan, Allan Wu, Guido C. Faas, Andrew C. Charles, Marco Iacoboni
