# Supplementary material for: Ultrasound stimulation of the motor cortex during tonic muscle contraction
Source: PLoS One. 2022 Apr 20;17(4):e0267268. doi: 10.1371/journal.pone.0267268 (PMC9020726; doi:10.1371/journal.pone.0267268)
Supplement: S9 Fig — Histograms and density plots shown by % aMT. Welch’s t-tests performed as post-hoc tests confirmed cSP duration increased by % aMT (p < 0.001, all pairs). For non-demeaned data see Fig 5 and S8 Fig. One subject (sbj08) with whom resting motor threshold was used is not shown here (see S8 Fig). (PDF) [file pone.0267268.s009.pdf]

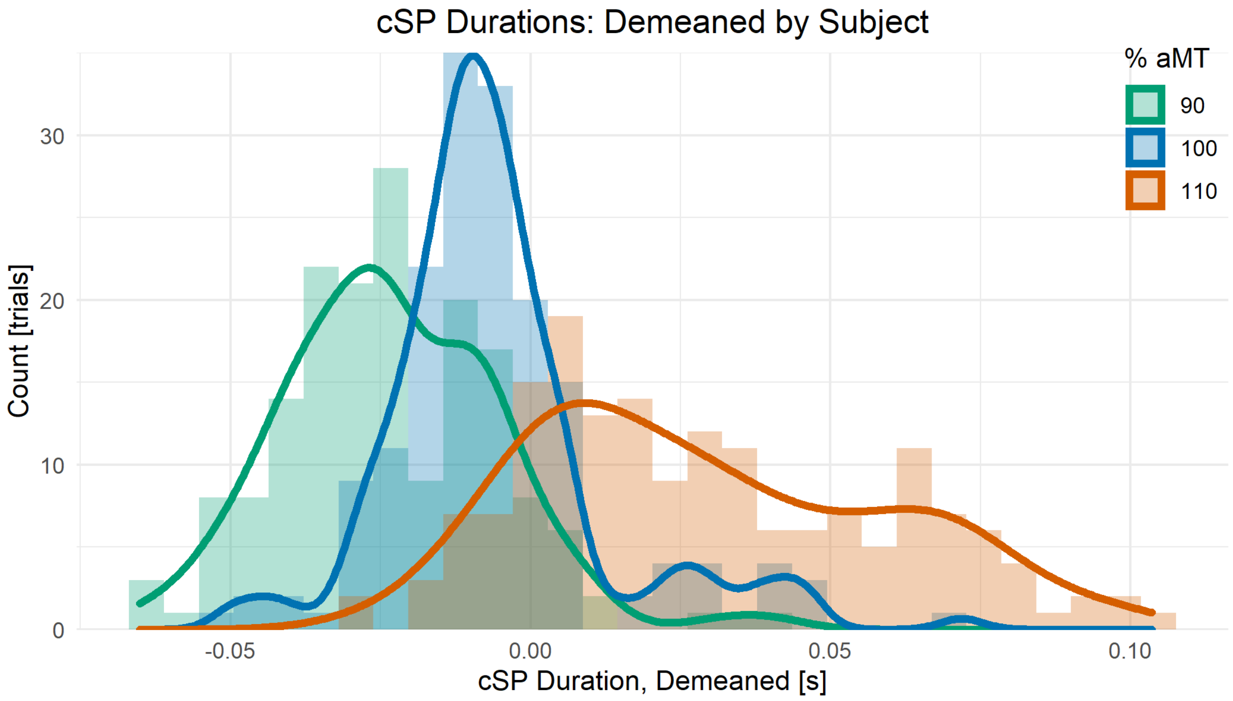

S9 Fig. cSP durations demeaned by subject mean. *Histograms and density plots shown by % aMT. Welch's t-tests performed as post-hoc tests confirmed cSP duration increased by % aMT (p < 0.001, all pairs). For non-demeaned data see Fig 5 and S8 Fig. One subject (sbj08) with whom resting motor threshold was used is not shown here (see S8 Fig).*
