# Supplementary material for: Ultrasound stimulation of the motor cortex during tonic muscle contraction
Source: PLoS One. 2022 Apr 20;17(4):e0267268. doi: 10.1371/journal.pone.0267268 (PMC9020726; doi:10.1371/journal.pone.0267268)
Supplement: S10 Fig — All three trials are from the same subject. The automated trial designation classified each trial (Top, Middle, Bottom) as:”cSP”,”Stub”,”Stub”. These two distinct examples of scenarios that fall under the “Stub” designation, as determined by the algorithm. This illustrates that there are likely two main distributions of trials that fall under the “Stub” designation. The first: trials in which there is a TMS-evoked MEP that is shorter than the standard threshold (0.5 mV). The second: trials in which there was by chance an EMG peak produced by tonic muscle contraction that fell within the expected time window. (PDF) [file pone.0267268.s010.pdf]

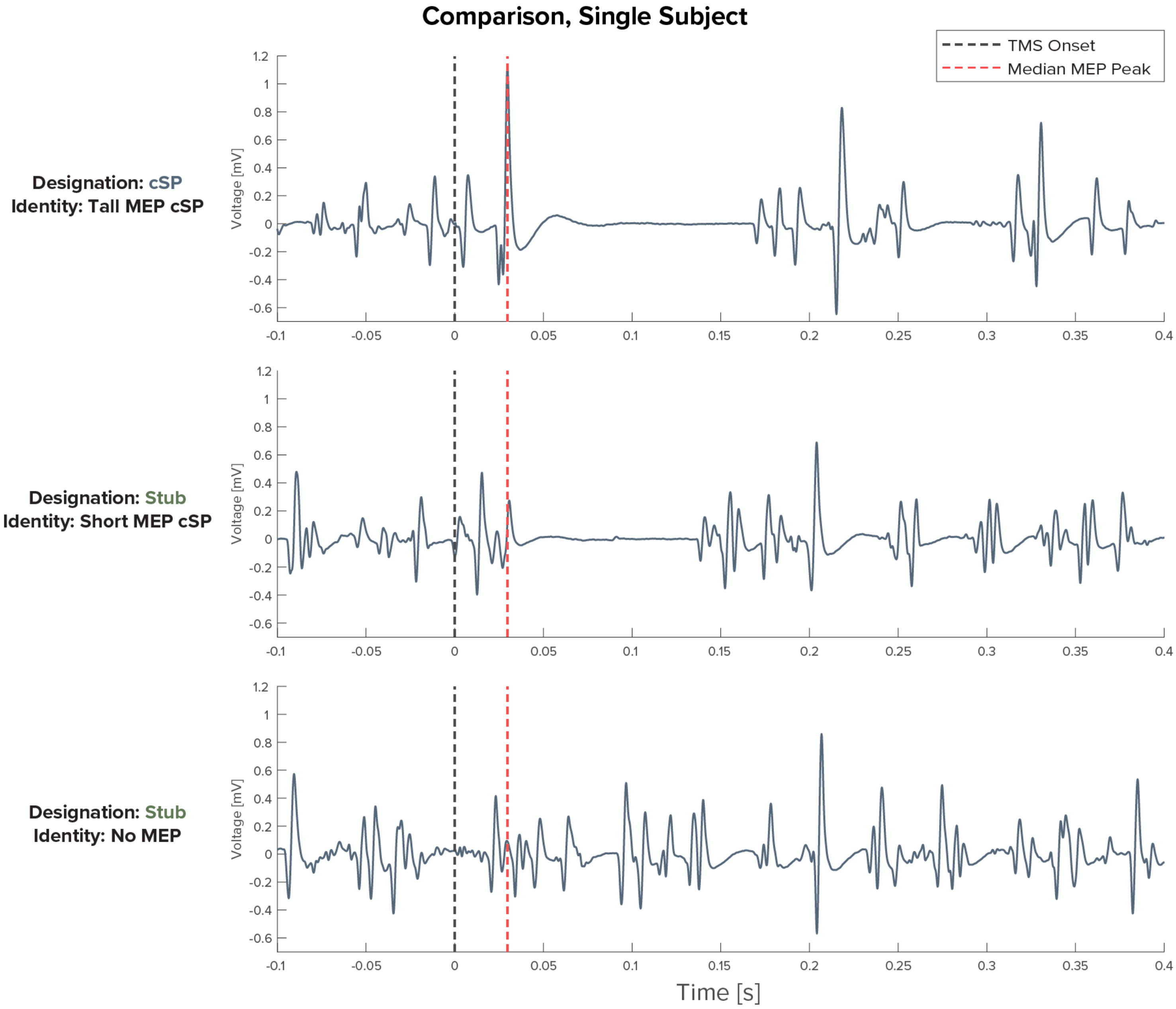

S10 Fig. Comparison of three different results from single-pulse TMS during tonic contraction. *All three trials are from the same subject. The automated trial designation classified each trial (**Top**, **Middle**, **Bottom**) as: "cSP", "Stub", "Stub". These two distinct examples of scenarios that fall under the "Stub" designation, as determined by the algorithm. This illustrates that there are likely two main distributions of trials that fall under the "Stub" designation. The first: trials in which there is a TMS-evoked MEP that is shorter than the standard threshold (0.5 mV). The second: trials in which there was by chance an EMG peak produced by tonic muscle contraction that fell within the expected time window.*

Supporting information for:

Ultrasound stimulation of the motor cortex during tonic muscle contraction

Ian S. Heimbuch, Tiffany K. Fan, Allan Wu, Guido C. Faas, Andrew C. Charles, Marco Iacoboni
