# Supplementary material for: Ultrasound stimulation of the motor cortex during tonic muscle contraction
Source: PLoS One. 2022 Apr 20;17(4):e0267268. doi: 10.1371/journal.pone.0267268 (PMC9020726; doi:10.1371/journal.pone.0267268)
Supplement: S11 Fig — TMS onset at 0 s. “None” trials had no prominent EMG peak within the 10-ms search window. Peaks had to be above the 50th percentile for peak prominence and above the 50th percentile for peak width (for EMG peaks within the 1-second trial). (PDF) [file pone.0267268.s011.pdf]

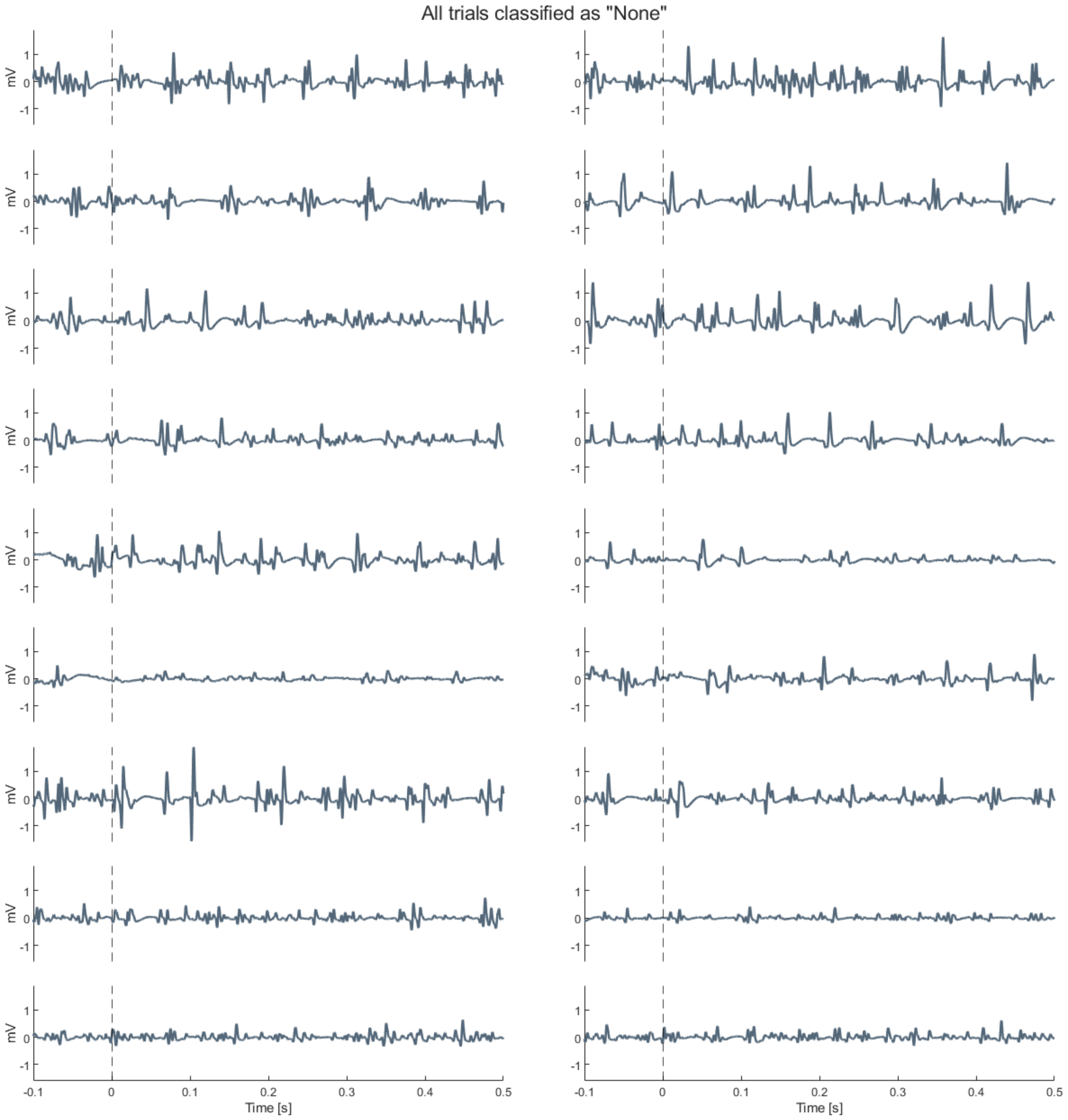

S11 Fig. All tonic contraction TMS trials designated as "None". *TMS onset at 0 s. "None" trials had no prominent EMG peak within the 10-ms search window. Peaks had to be above the 50th percentile for peak prominence and above the 50th percentile for peak width (for EMG peaks within the 1-second trial).*

Supporting information for:

Ultrasound stimulation of the motor cortex during tonic muscle contraction

Ian S. Heimbuch, Tiffany K. Fan, Allan Wu, Guido C. Faas, Andrew C. Charles, Marco Iacoboni
