# Supplementary material for: Ultrasound stimulation of the motor cortex during tonic muscle contraction
Source: PLoS One. 2022 Apr 20;17(4):e0267268. doi: 10.1371/journal.pone.0267268 (PMC9020726; doi:10.1371/journal.pone.0267268)
Supplement: S12 Fig — Illustration of the TMS search grid used in both 2D and 3D. The grid’s origin (white) was placed at MNI coordinates that correspond to M1hand as based on a meta-analysis of fMRI motor experiments: x = −39, y = −24, z = 57 [43]. The other five targets on the grid (grey) were in a 12 voxel-width grid (9.6 mm grid interval) around M1hand in subject space. See EMG and NIBS Placement. (PDF) [file pone.0267268.s012.pdf]

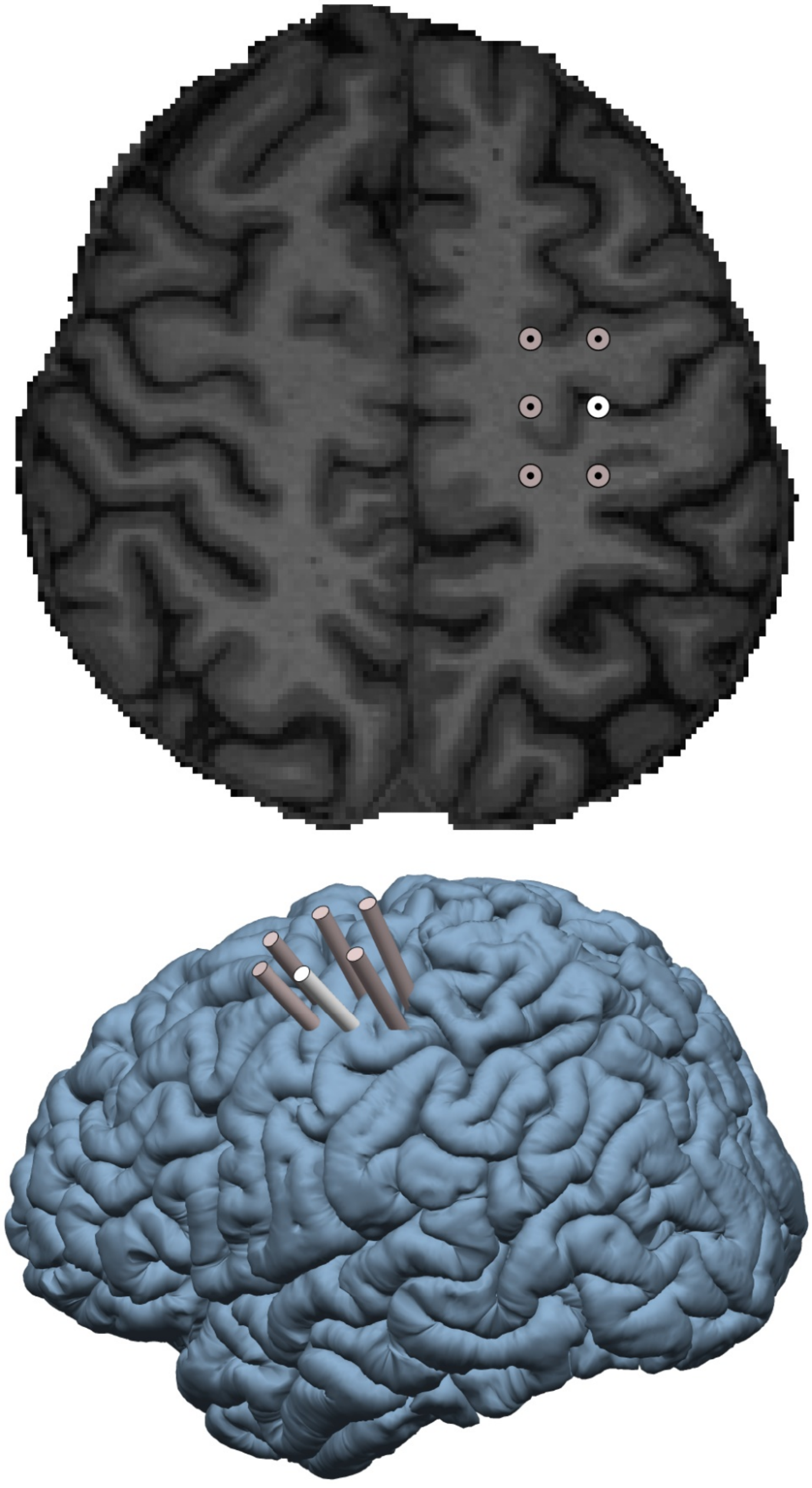

S12 Fig. TMS search grid and trajectories. Illustration of the TMS search grid used in both 2D and 3D. *The grid's origin (white) was placed at MNI coordinates that correspond to* $M1_{hand}$ *as based on a meta-analysis of fMRI motor experiments:* $x = -39$, $y = -24$, $z = 57$ [45]. *The other five targets on the grid (grey) were in a 12 voxel-width grid (9.6 mm grid interval) around* $M1_{hand}$ *in subject space. See EMG and NIBS Placement.*

Supporting information for:

Ultrasound stimulation of the motor cortex during tonic muscle contraction
Ian S. Heimbuch, Tiffany K. Fan, Allan Wu, Guido C. Faas, Andrew C. Charles, Marco Iacoboni
