# Supplementary figures and images for: Ultrasound stimulation of the motor cortex during tonic muscle contraction
Source: PLoS One. 2022 Apr 20;17(4):e0267268. doi: 10.1371/journal.pone.0267268 (PMC9020726; doi:10.1371/journal.pone.0267268)

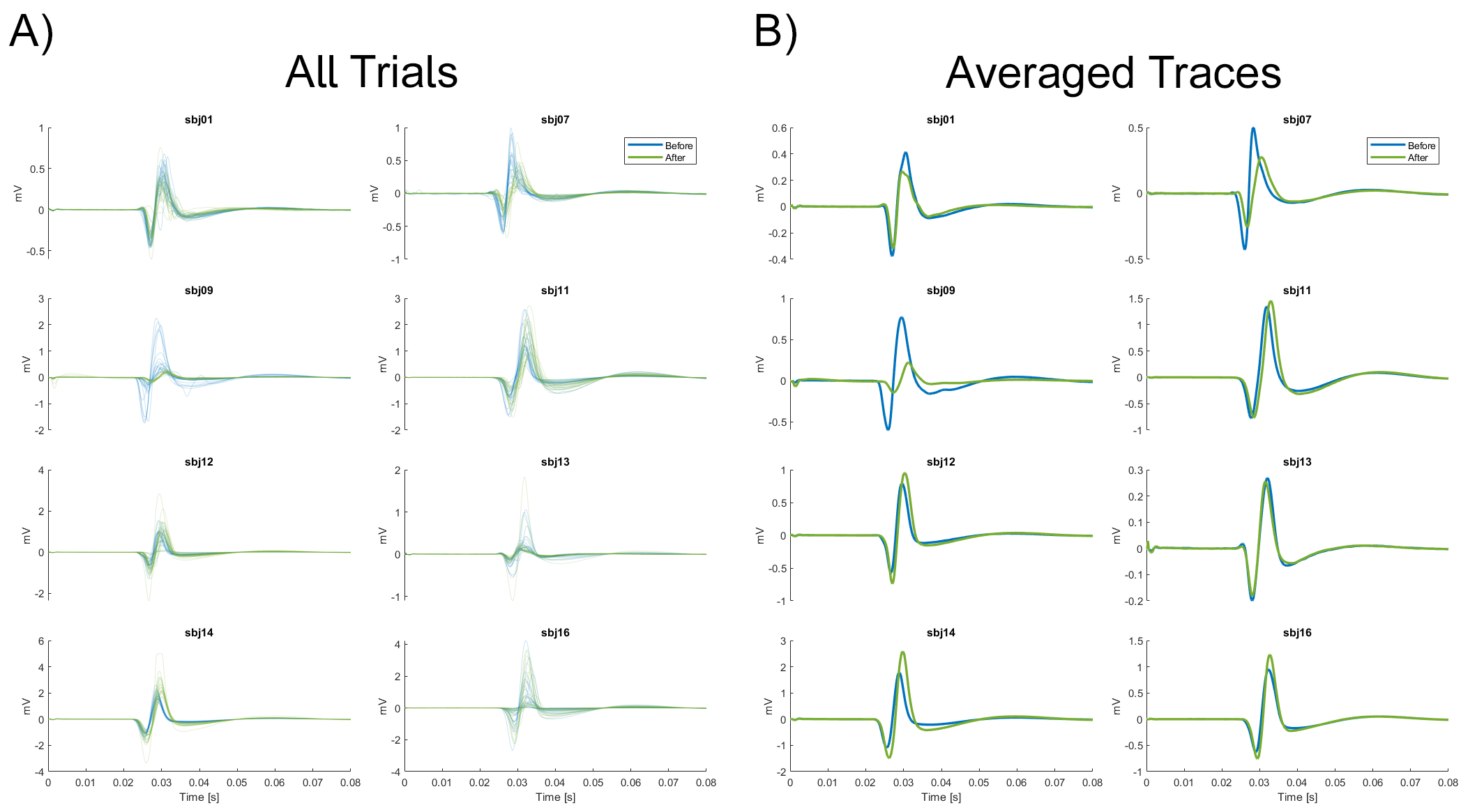

Supplement: S13 Fig — MEP waveforms for Experiment 2 for each subject. MEPs measured before (blue) and after (green) tUS exposure. (a) All waveforms. (b) Averaged waveforms. (TIF) [file pone.0267268.s013.tif]

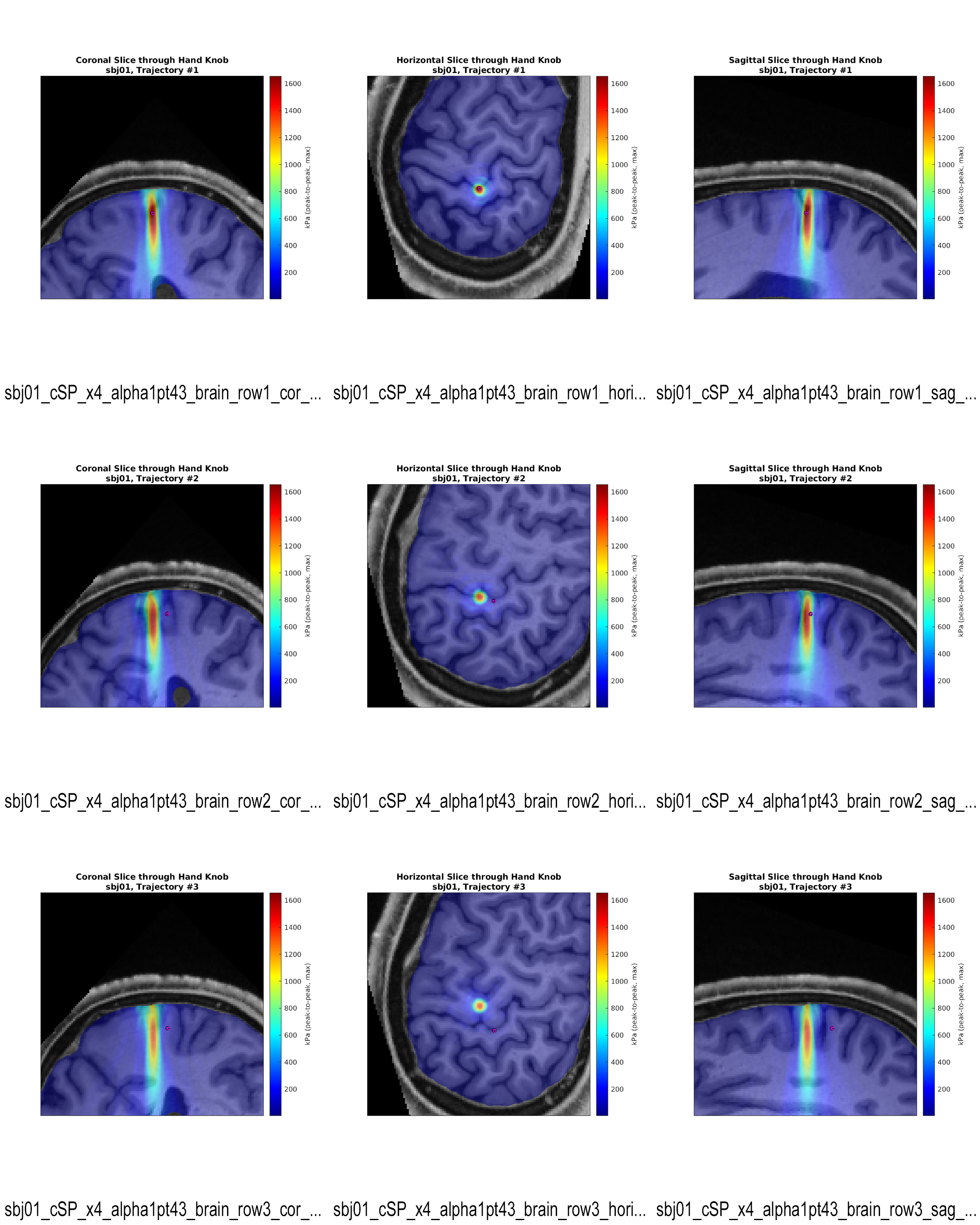

Supplement: S14 Fig — Simulated pressure maps are overlayed over the respective structural MRI. One file per subject. One target per row. 3 slices per target. Slices shown at the maximum pressure value. Note: these are not standard slices (i.e. coronal, sagittal, horizontal), since the volume was reoriented as part of pre-simulation processing, A small magenta circle denotes the registered M1 coordinate. (ZIP) [file pone.0267268.s014.zip › sbj01_cSP_x4_alpha1pt43_brain_matchedAllSbjLimits_proofSheet.png]

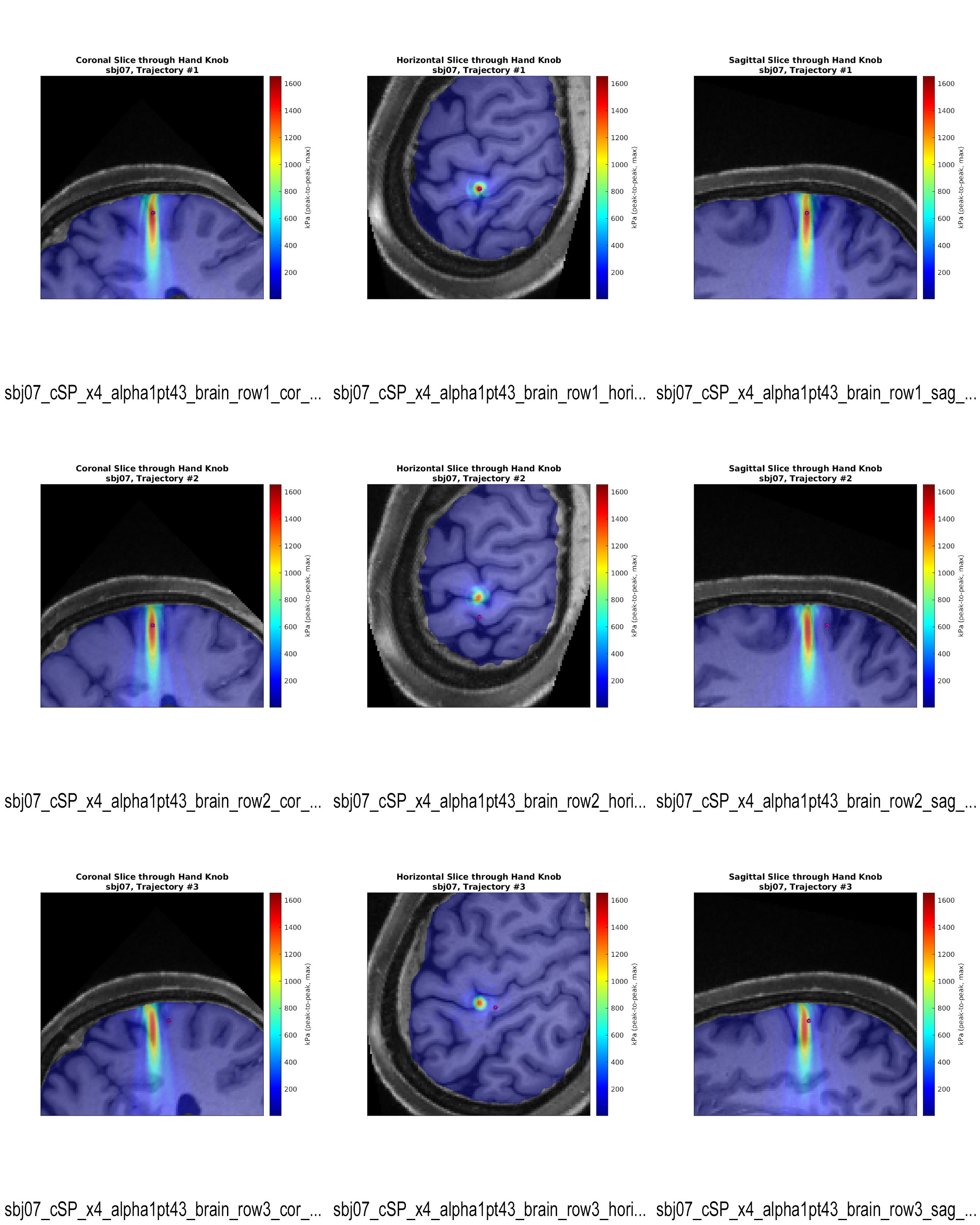

Supplement: S14 Fig — Simulated pressure maps are overlayed over the respective structural MRI. One file per subject. One target per row. 3 slices per target. Slices shown at the maximum pressure value. Note: these are not standard slices (i.e. coronal, sagittal, horizontal), since the volume was reoriented as part of pre-simulation processing, A small magenta circle denotes the registered M1 coordinate. (ZIP) [file pone.0267268.s014.zip › sbj07_cSP_x4_alpha1pt43_brain_matchedAllSbjLimits_proofSheet.png]

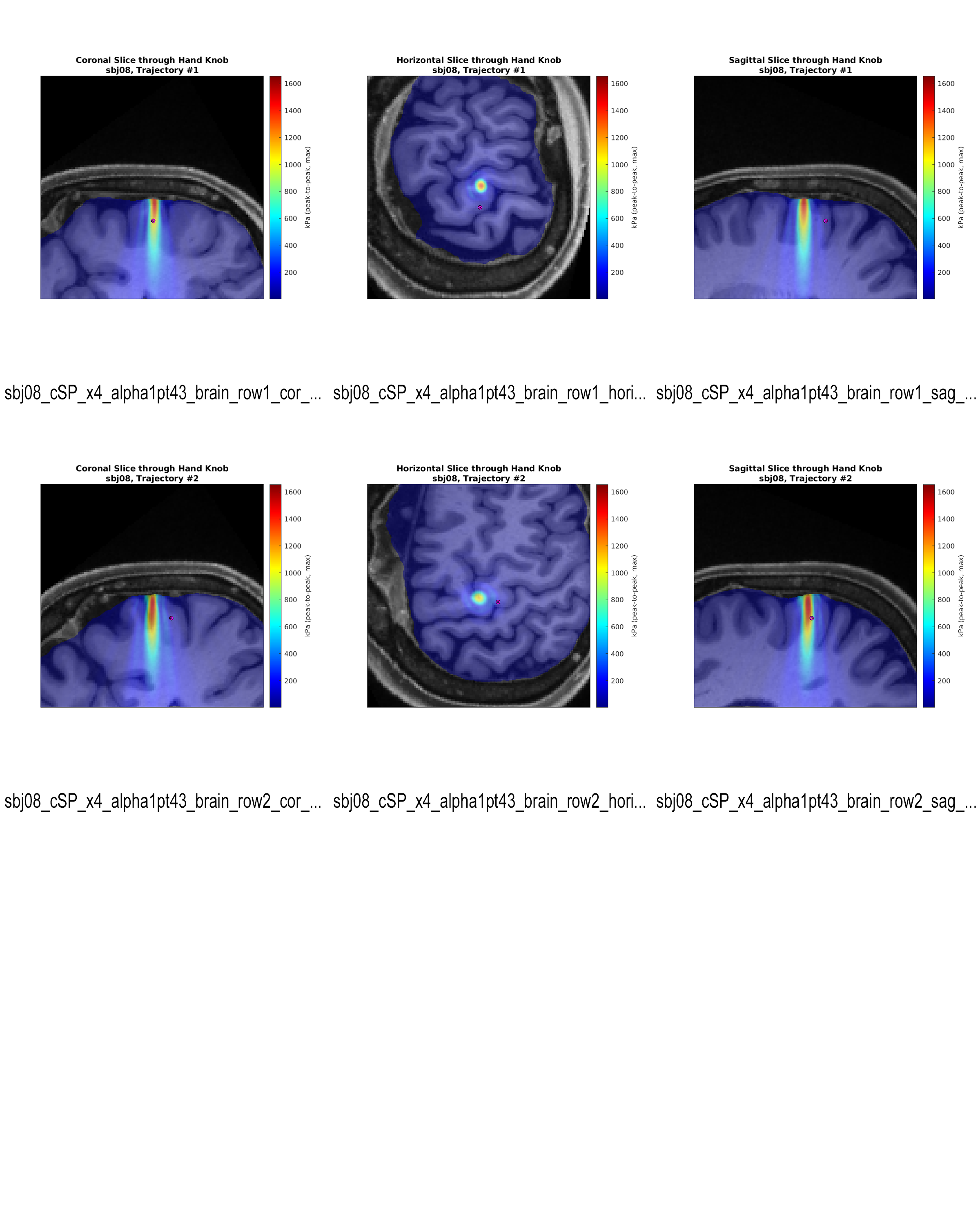

Supplement: S14 Fig — Simulated pressure maps are overlayed over the respective structural MRI. One file per subject. One target per row. 3 slices per target. Slices shown at the maximum pressure value. Note: these are not standard slices (i.e. coronal, sagittal, horizontal), since the volume was reoriented as part of pre-simulation processing, A small magenta circle denotes the registered M1 coordinate. (ZIP) [file pone.0267268.s014.zip › sbj08_cSP_x4_alpha1pt43_brain_matchedAllSbjLimits_proofSheet.png]

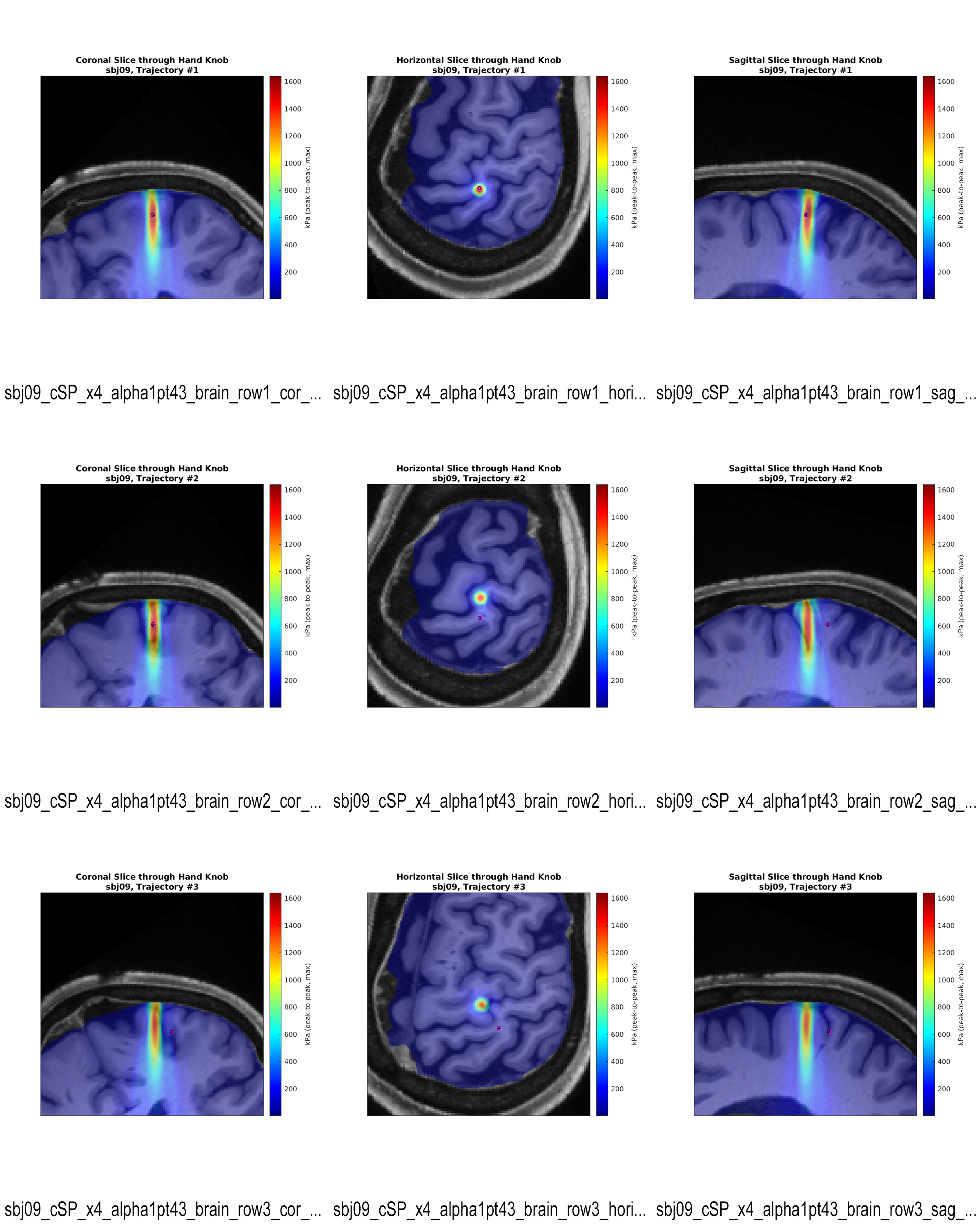

Supplement: S14 Fig — Simulated pressure maps are overlayed over the respective structural MRI. One file per subject. One target per row. 3 slices per target. Slices shown at the maximum pressure value. Note: these are not standard slices (i.e. coronal, sagittal, horizontal), since the volume was reoriented as part of pre-simulation processing, A small magenta circle denotes the registered M1 coordinate. (ZIP) [file pone.0267268.s014.zip › sbj09_cSP_x4_alpha1pt43_brain_matchedAllSbjLimits_proofSheet.png]

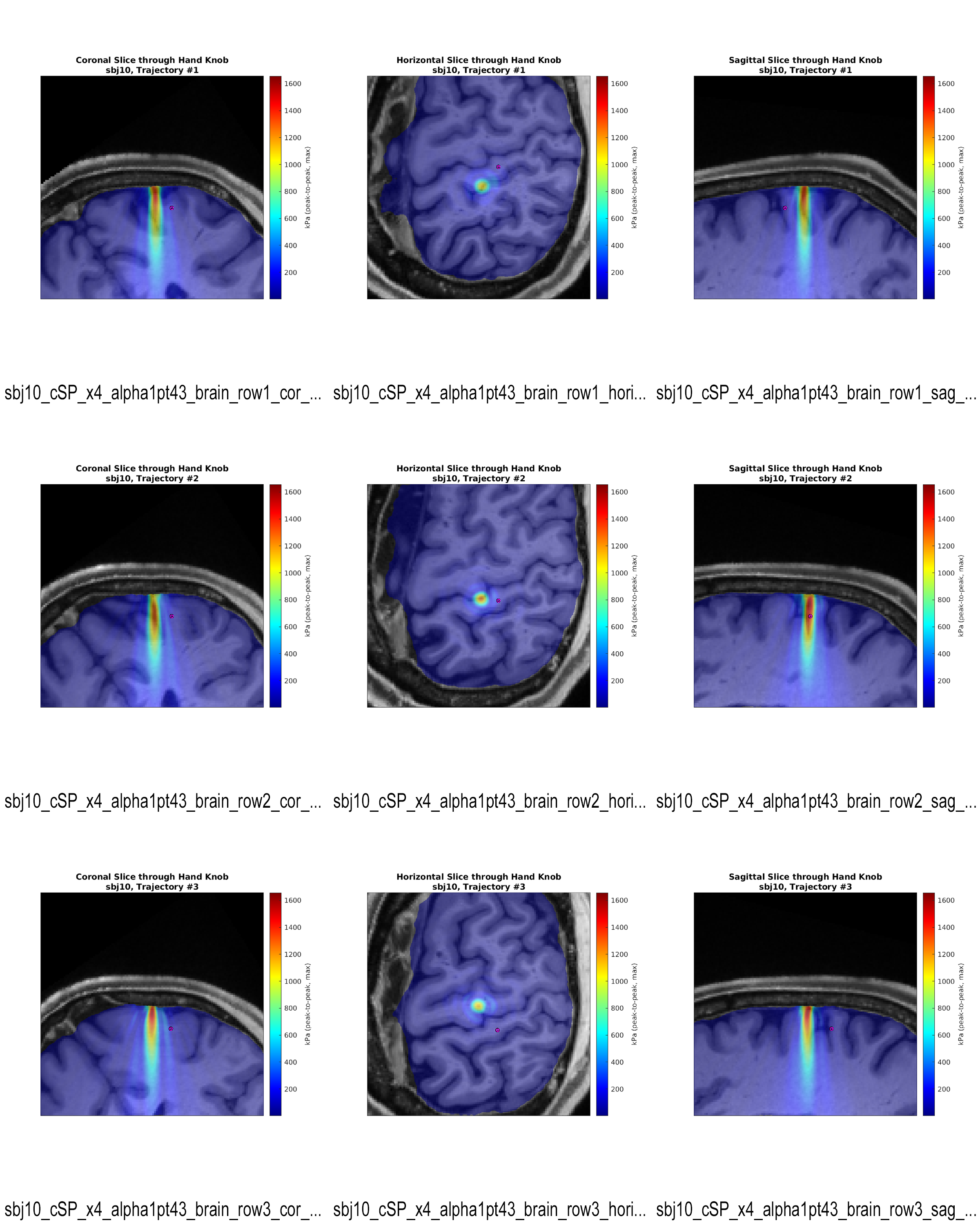

Supplement: S14 Fig — Simulated pressure maps are overlayed over the respective structural MRI. One file per subject. One target per row. 3 slices per target. Slices shown at the maximum pressure value. Note: these are not standard slices (i.e. coronal, sagittal, horizontal), since the volume was reoriented as part of pre-simulation processing, A small magenta circle denotes the registered M1 coordinate. (ZIP) [file pone.0267268.s014.zip › sbj10_cSP_x4_alpha1pt43_brain_matchedAllSbjLimits_proofSheet.png]

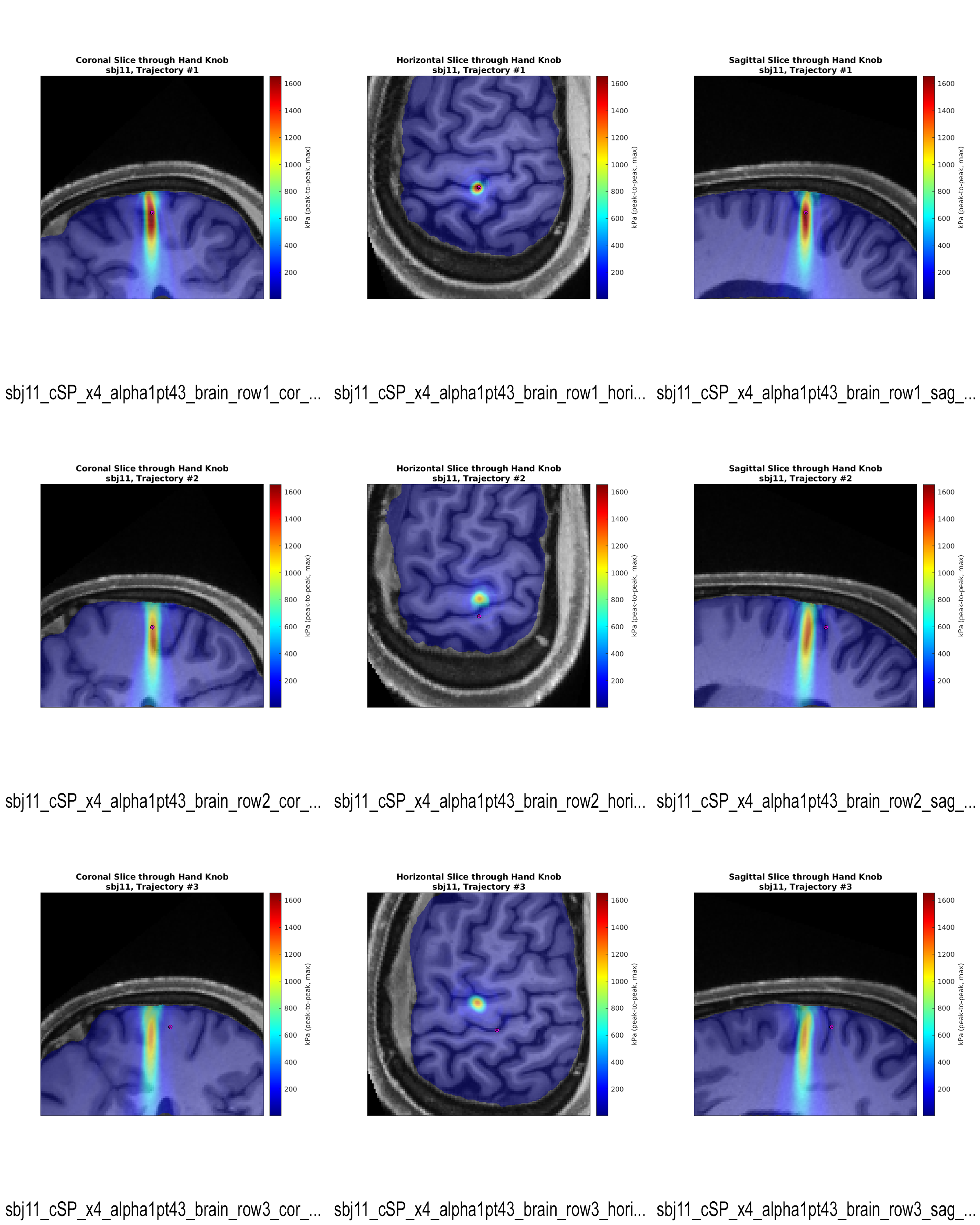

Supplement: S14 Fig — Simulated pressure maps are overlayed over the respective structural MRI. One file per subject. One target per row. 3 slices per target. Slices shown at the maximum pressure value. Note: these are not standard slices (i.e. coronal, sagittal, horizontal), since the volume was reoriented as part of pre-simulation processing, A small magenta circle denotes the registered M1 coordinate. (ZIP) [file pone.0267268.s014.zip › sbj11_cSP_x4_alpha1pt43_brain_matchedAllSbjLimits_proofSheet.png]

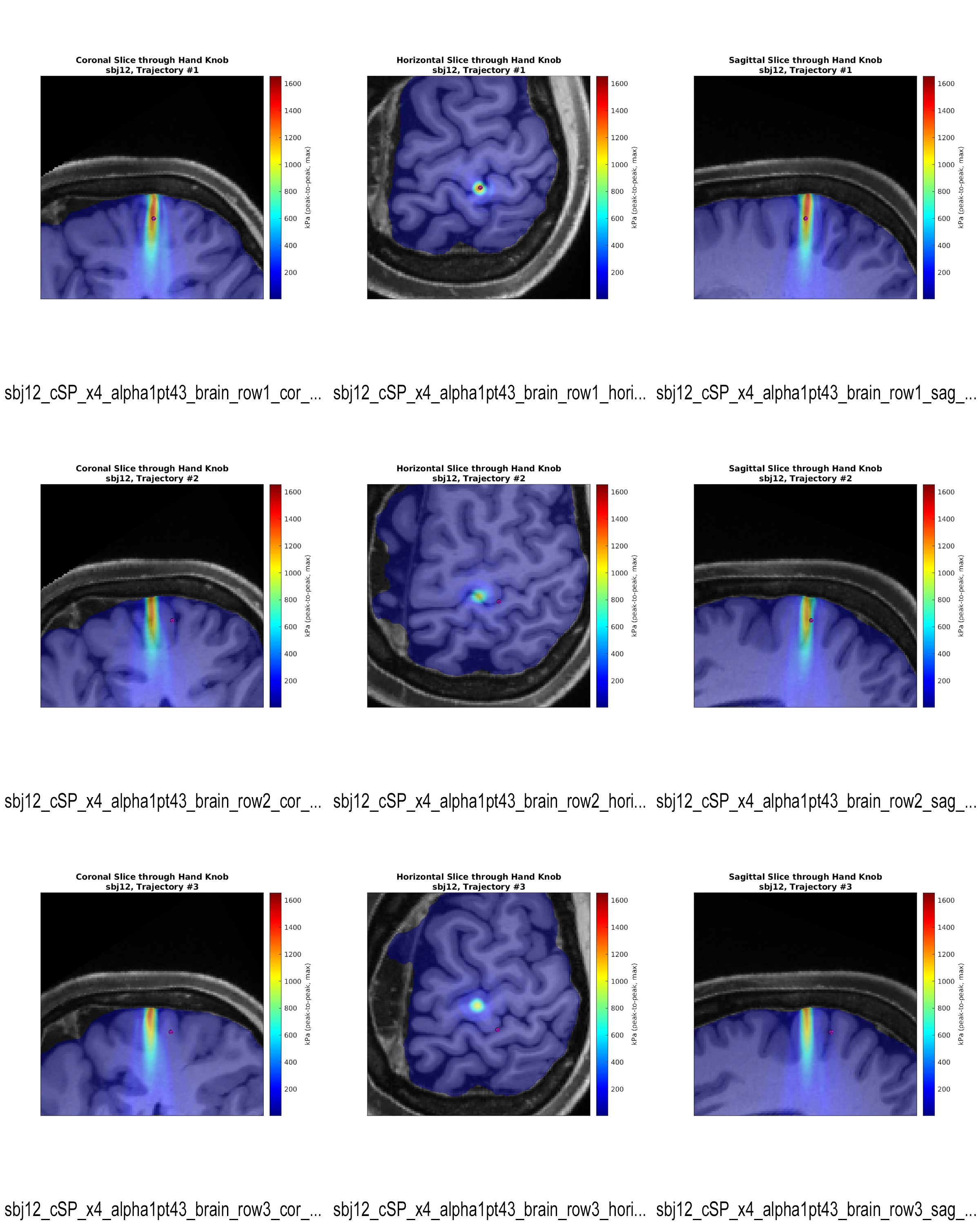

Supplement: S14 Fig — Simulated pressure maps are overlayed over the respective structural MRI. One file per subject. One target per row. 3 slices per target. Slices shown at the maximum pressure value. Note: these are not standard slices (i.e. coronal, sagittal, horizontal), since the volume was reoriented as part of pre-simulation processing, A small magenta circle denotes the registered M1 coordinate. (ZIP) [file pone.0267268.s014.zip › sbj12_cSP_x4_alpha1pt43_brain_matchedAllSbjLimits_proofSheet.png]

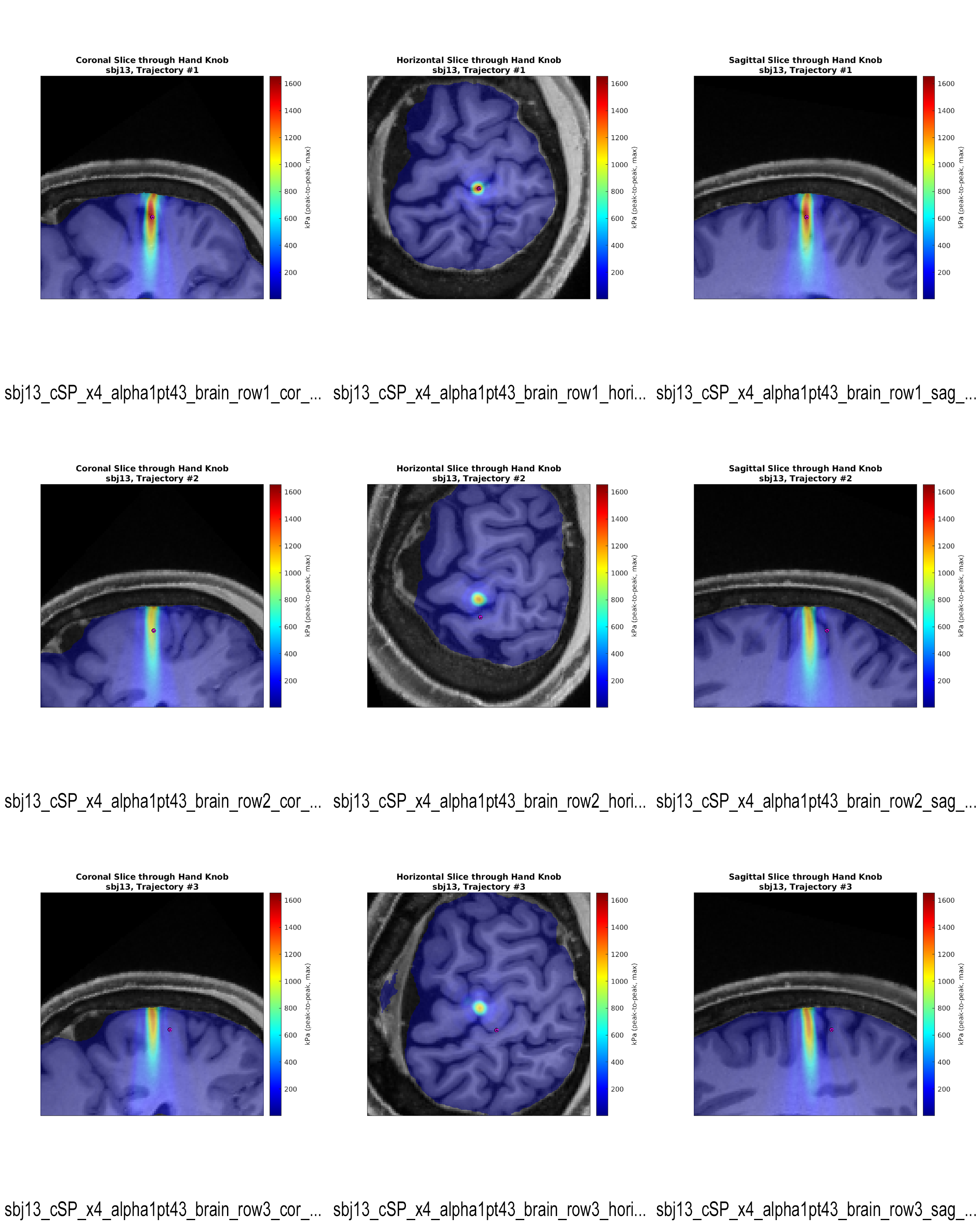

Supplement: S14 Fig — Simulated pressure maps are overlayed over the respective structural MRI. One file per subject. One target per row. 3 slices per target. Slices shown at the maximum pressure value. Note: these are not standard slices (i.e. coronal, sagittal, horizontal), since the volume was reoriented as part of pre-simulation processing, A small magenta circle denotes the registered M1 coordinate. (ZIP) [file pone.0267268.s014.zip › sbj13_cSP_x4_alpha1pt43_brain_matchedAllSbjLimits_proofSheet.png]

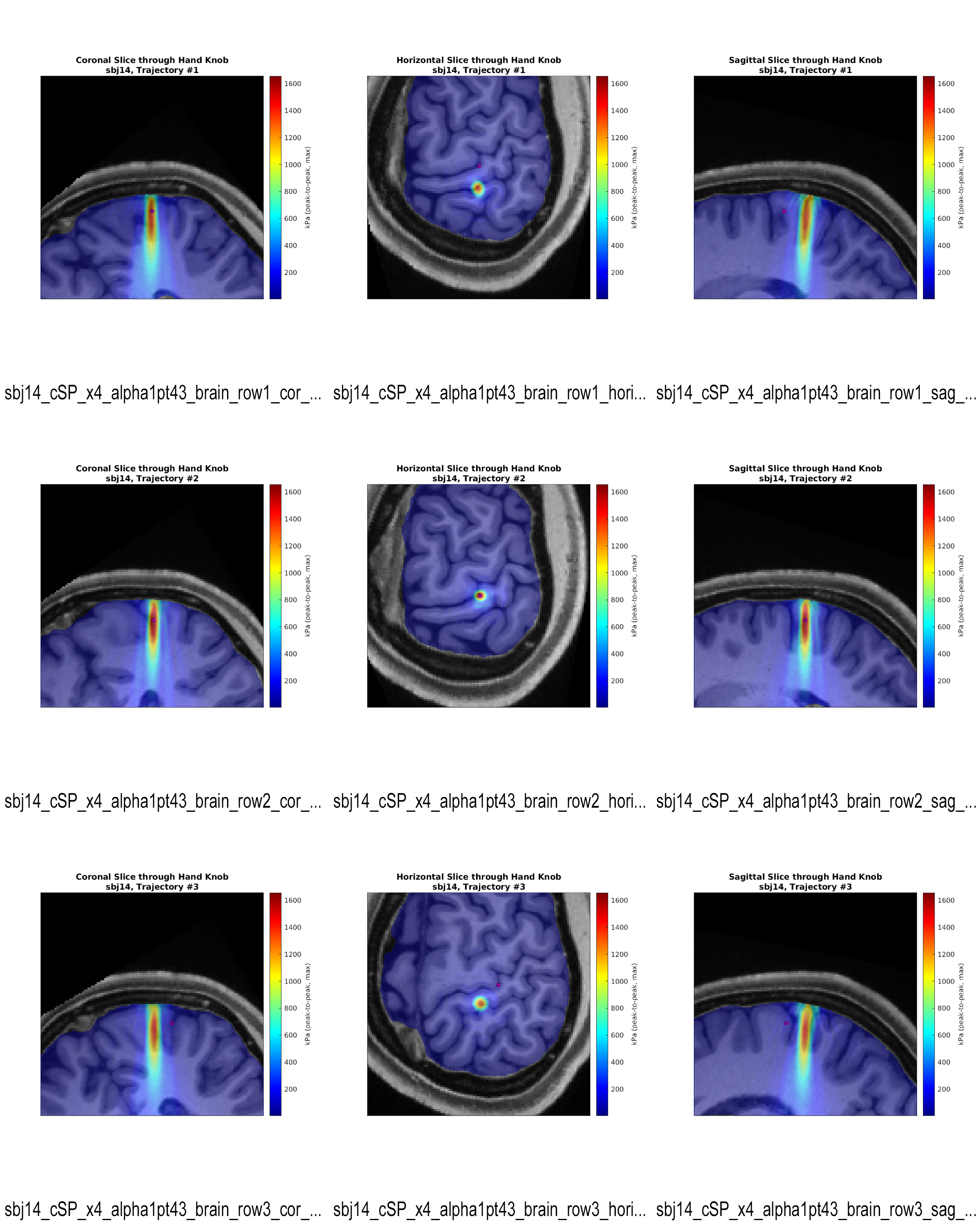

Supplement: S14 Fig — Simulated pressure maps are overlayed over the respective structural MRI. One file per subject. One target per row. 3 slices per target. Slices shown at the maximum pressure value. Note: these are not standard slices (i.e. coronal, sagittal, horizontal), since the volume was reoriented as part of pre-simulation processing, A small magenta circle denotes the registered M1 coordinate. (ZIP) [file pone.0267268.s014.zip › sbj14_cSP_x4_alpha1pt43_brain_matchedAllSbjLimits_proofSheet.png]

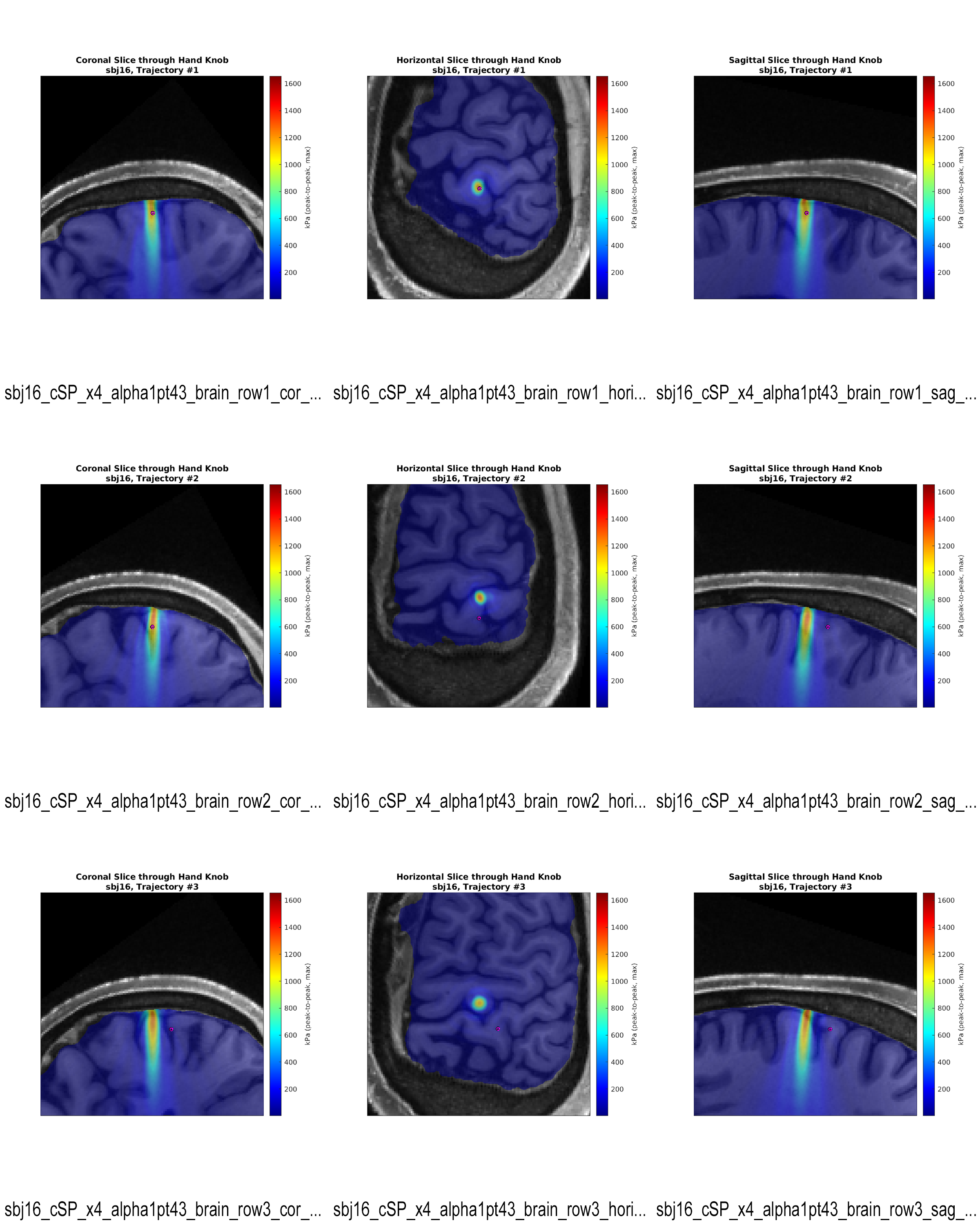

Supplement: S14 Fig — Simulated pressure maps are overlayed over the respective structural MRI. One file per subject. One target per row. 3 slices per target. Slices shown at the maximum pressure value. Note: these are not standard slices (i.e. coronal, sagittal, horizontal), since the volume was reoriented as part of pre-simulation processing, A small magenta circle denotes the registered M1 coordinate. (ZIP) [file pone.0267268.s014.zip › sbj16_cSP_x4_alpha1pt43_brain_matchedAllSbjLimits_proofSheet.png]

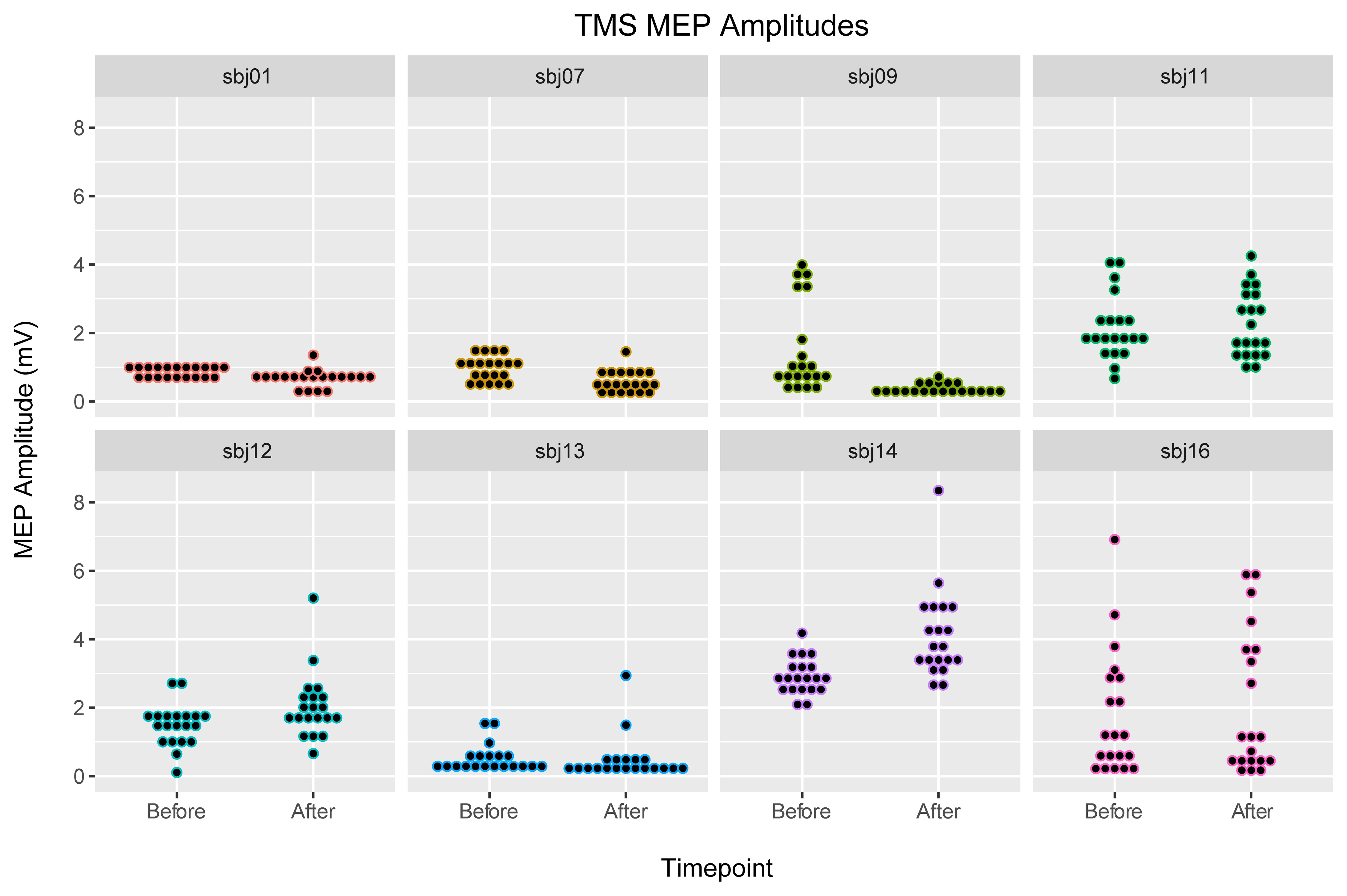

Supplement: S15 Fig — Each subplot contains data from one subject: trials before and after tUS on the left and right, respectively. Each point marks one MEP amplitude (mV). Points are organized into vertical-axis bins to aid in visualization. Delta: 0.1 mV; 95% CI: -0.32, 0.29 mV; p = 0.17. No significant difference was found when using area under the curve, as well (Fig 5). (TIF) [file pone.0267268.s015.tif]

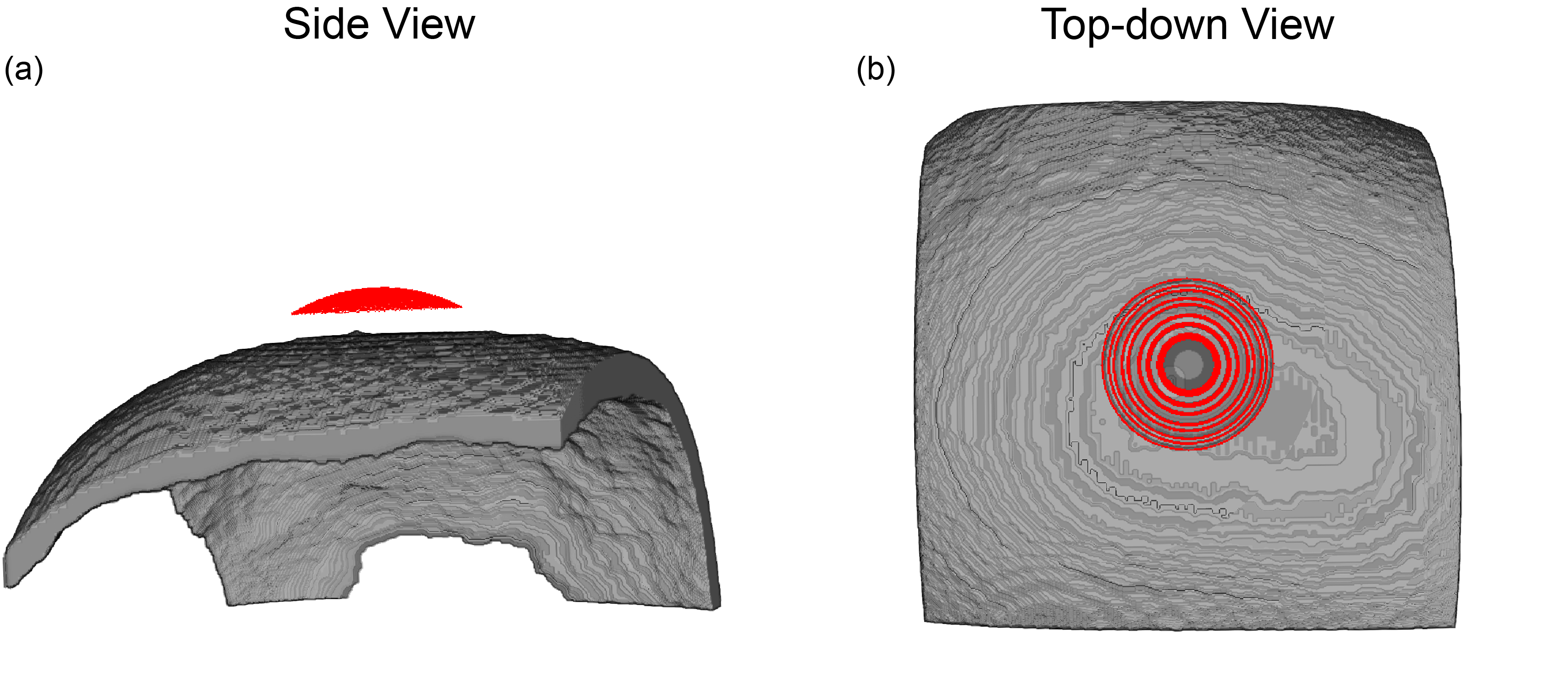

Supplement: S16 Fig — A 3D render of the volumetric data from one example simulation to show the position of the transducer (red) relative to the skull (grey). Same render viewed from (a) the side and (b) the top down. Images show that neither the transducer nor critical portions of the skull mask were cut off by cropping done during preprocessing prior to simulation. Volumetric (voxel) data was from the transducer map (starting pressure binary map) and medium data output by k-Wave. (PNG) [file pone.0267268.s016.png]

## Examples of skull masks overlaid onto T1

**sbj01**  
Trajectory 1

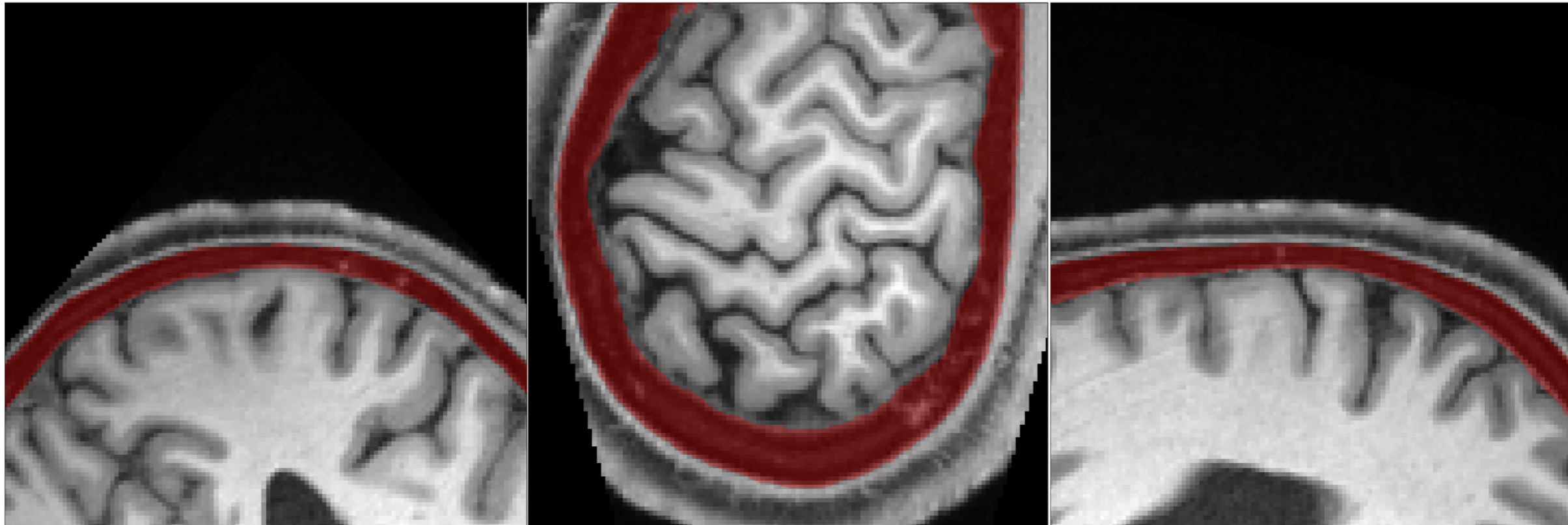

**sbj11**  
Trajectory 1

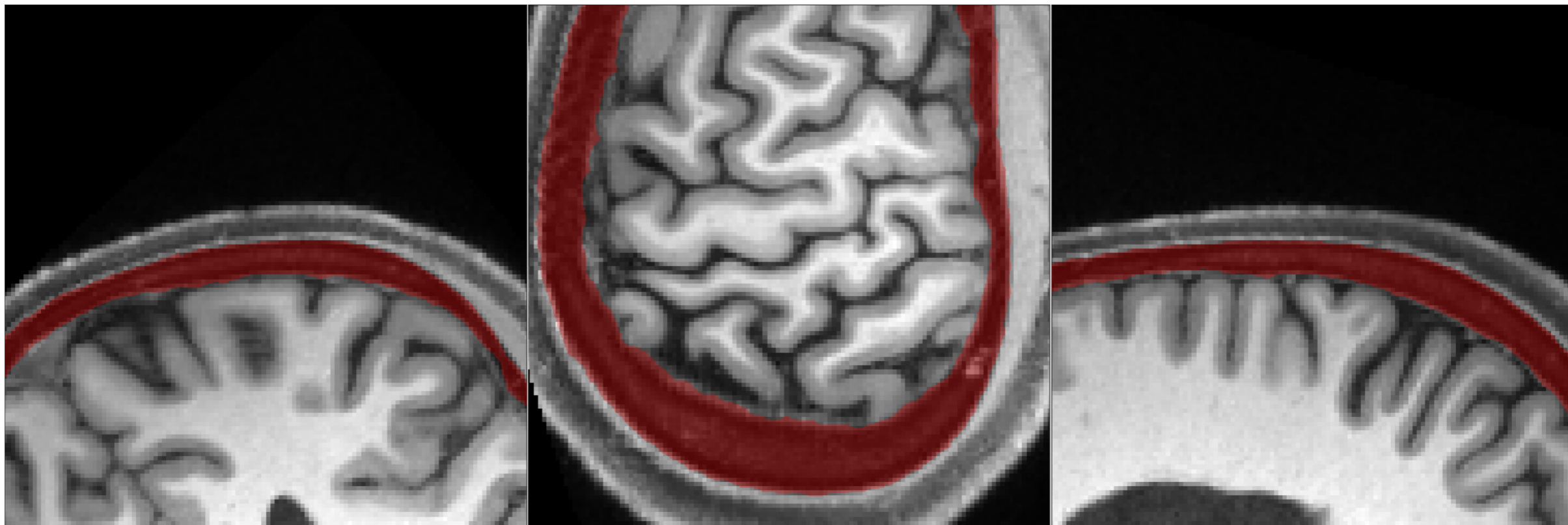

**sbj01**  
Trajectory 3

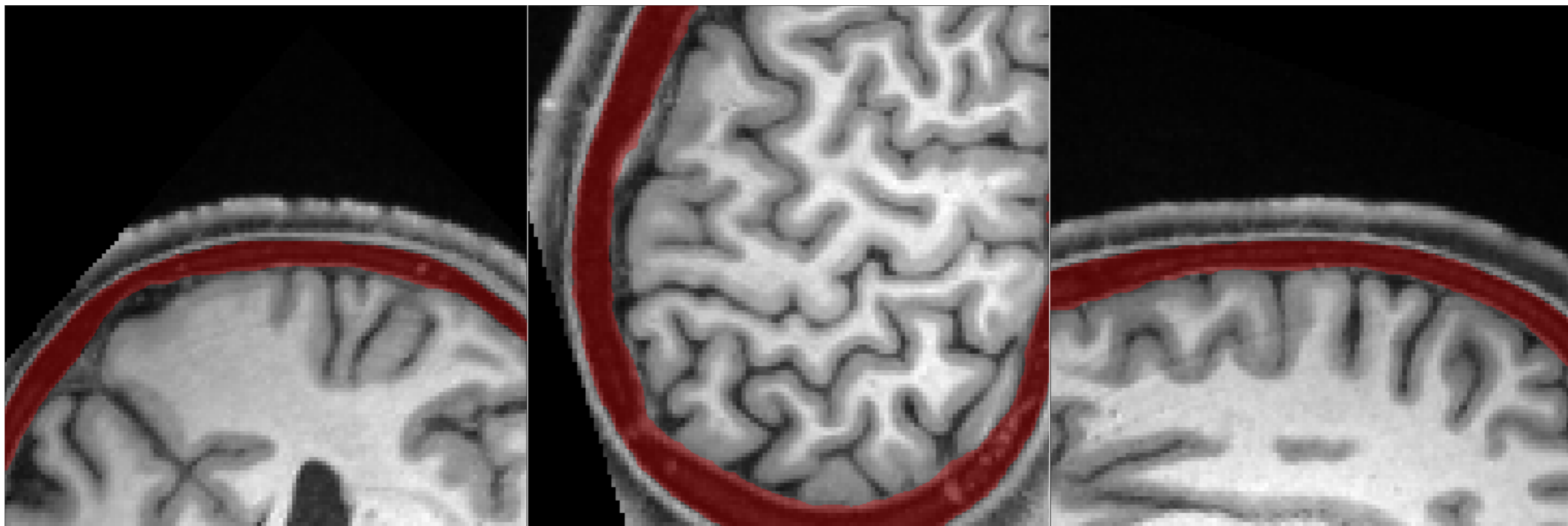

Supplement: S17 Fig — Examples of skull masks used in acoustic simulation overlayed onto their respective subject-specific structural MRIs. Slices are the same as those shown in Fig 7. Masks shown are those used during simulation (i.e. after full skull processing including upscaling and smoothing via morphological image processing). Slices shown were made at the maximum pressure value (as visible in Fig 7). Note that these are not standard slices (i.e. coronal, sagittal, horizontal), since the volume was reoriented as part of pre-simulation processing. (PDF) [file pone.0267268.s017.pdf]
