# Supplementary material for: Ultrasound stimulation of the motor cortex during tonic muscle contraction
Source: PLoS One. 2022 Apr 20;17(4):e0267268. doi: 10.1371/journal.pone.0267268 (PMC9020726; doi:10.1371/journal.pone.0267268)
Supplement: S18 Fig — An illustration of the trajectory search protocol and tUS exposure protocol shared by both Experiment 1 and Experiment 2. (a) The search protocol used to find which three trajectories to use for tUS within the search grid (S12 Fig). For a search session (illustrative data), the mean TMS MEP trace generated at each trajectory is shown next to its corresponding grid location. The targets that elicited the largest, second-largest, and third-largest average MEPs were used as placement points for the NIBS devices. These three positions are referred to as “TMS target” (blue box), “2nd-best” (yellow box), and “3rd-best” (green box) targets respectively. (b) Illustration of the tUS burst durations per tUS trajectory. For full tUS protocol, see Fig 1. (PDF) [file pone.0267268.s018.pdf]

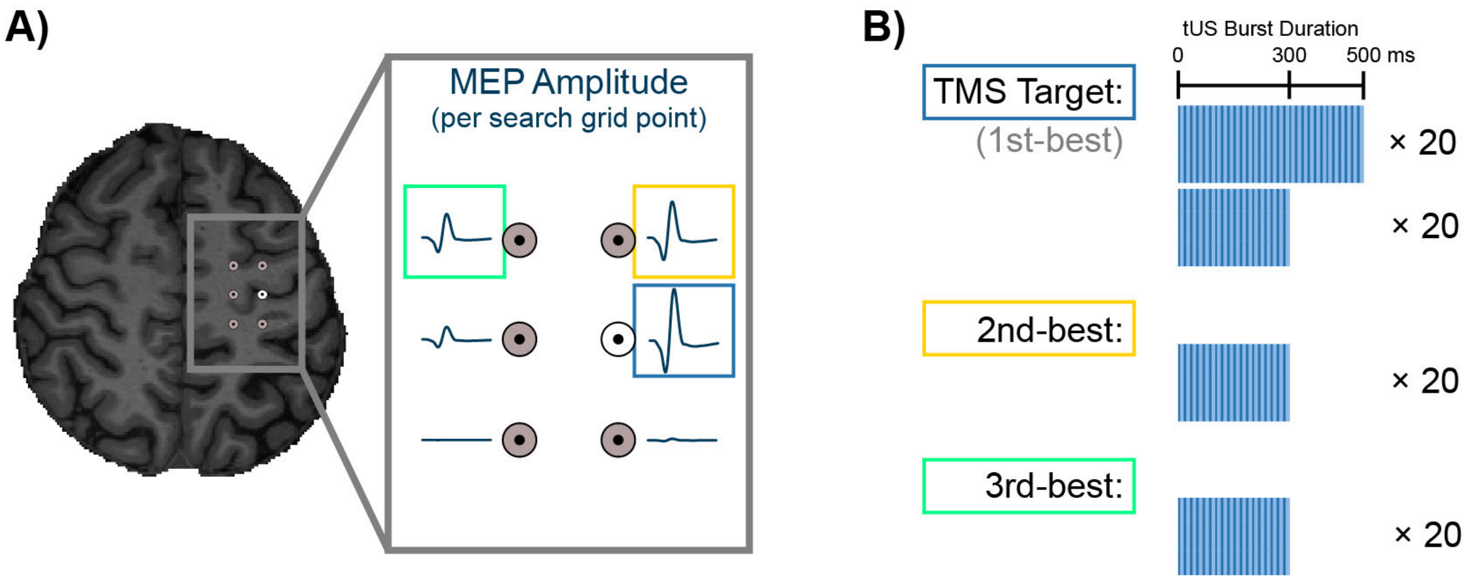

S22 Fig. tUS trajectory choice and tUS exposure protocol (location). An illustration of the trajectory search protocol and tUS exposure protocol shared by both Experiment 1 and Experiment 2. (a) The search protocol used to find which three trajectories to use for tUS within the search grid (*S12 Fig*). For a search session (illustrative data), the mean TMS MEP trace generated at each trajectory is shown next to its corresponding grid location. The targets that elicited the largest, second-largest, and third-largest average MEPs were used as placement points for the NIBS devices. These three positions are referred to as "TMS target" (blue box), "2nd-best" (yellow box), and "3rd-best" (green box) targets respectively. (b) Illustration of the tUS burst durations per tUS trajectory. For full tUS protocol, see ***Fig 1***.
