# Supplementary material for: Ultrasound stimulation of the motor cortex during tonic muscle contraction
Source: PLoS One. 2022 Apr 20;17(4):e0267268. doi: 10.1371/journal.pone.0267268 (PMC9020726; doi:10.1371/journal.pone.0267268)
Supplement: S19 Fig — Sequence of events for acquisition of resting TMS-evoked MEPs and tUS exposure. Not included in this diagram is the acquisition of TMS-evoked cSPs (TMS cSPs), which were acquired before all other experimental sections in all cases. (a) Timeline for the two volunteers who participated in only Experiment 1. The four blocks of 20 tUS trials each during tonic contraction paradigm: 300 ms at TMS target, 300 ms at 2nd-best, 300 ms at 3rd-best, 500 ms at TMS target. See Experiment 1, cSPs for full protocol. (b) Timeline for the eight volunteers who participated in both Experiment 1 and Experiment 2. 20 TMS-evoked MEPs were acquired both before and after the tUS exposure protocol. See Experiment 2, Cortical excitability for full protocol. (PDF) [file pone.0267268.s019.pdf]

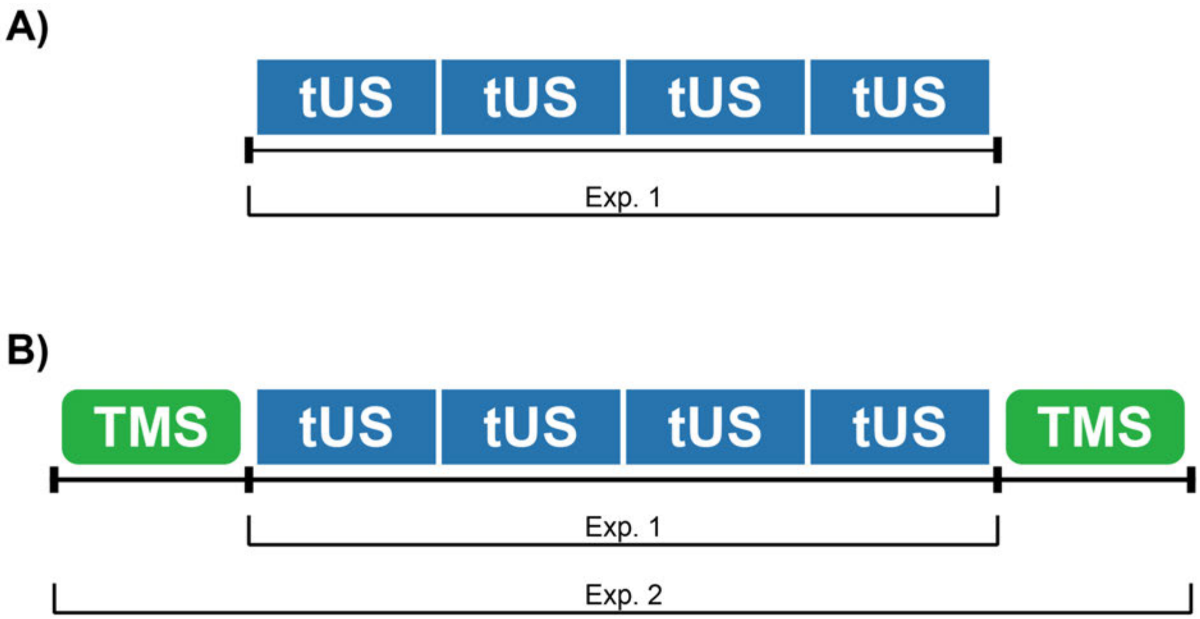

S23 Fig. Participant NIBS visit timelines. Sequence of events for acquisition of resting TMS-evoked MEPs and tUS exposure. Not included in this diagram is the acquisition of TMS-evoked cSPs (*TMS cSPs*), which were acquired before all other experimental sections in all cases. (a) Timeline for the two volunteers who participated in only Experiment 1. The four blocks of 20 tUS trials each during tonic contraction paradigm: 300 ms at TMS target, 300 ms at 2nd-best, 300 ms at 3rd-best, 500 ms at TMS target. See *Experiment 1, cSPs* for full protocol. (b) Timeline for the eight volunteers who participated in both Experiment 1 and Experiment 2. 20 TMS-evoked MEPs were acquired both before and after the tUS exposure protocol. See *Experiment 2, Cortical excitability* for full protocol.
