# Supplementary material for: Ultrasound stimulation of the motor cortex during tonic muscle contraction
Source: PLoS One. 2022 Apr 20;17(4):e0267268. doi: 10.1371/journal.pone.0267268 (PMC9020726; doi:10.1371/journal.pone.0267268)
Supplement: S1 Table — Data for both experiments included. Included are full width half maximum (FWHM) values [mm] of width of the ellipsoid focus of the focused ultrasound beam. The maximum pressures for three key locations for the simulation are also shown: the maximum pressure anywhere, at M1hand, and at the target coordinate used to aim the trajectory. In some cases, the trajectory coordinate and the M1hand coordinate are the same (‘Target’ column). (PDF) [file pone.0267268.s020.pdf]

Subject	FWHM [mm]			Pressure$_{Peak\text{-}to\text{-}Peak}$ [kPa]			Target
	Average	Dim. 1	Dim. 2	Anywhere	M1$_{hand}$	Target	
sbj01	4.4	4.4	4.4	470	441	441	M1$_{hand}$
sbj01	4.6	4.8	4.4	441	37	399	other
sbj01	4.9	5.0	4.8	406	19	378	other
sbj07	4.5	4.4	4.6	428	386	386	M1$_{hand}$
sbj07	4.3	4.4	4.2	451	23	375	other
sbj07	4.5	4.6	4.4	423	42	376	other
sbj08	4.2	4.0	4.4	418	18	357	other
sbj08	4.0	3.8	4.2	441	21	306	other
sbj09	4.5	4.6	4.4	463	438	446	M1$_{hand}$
sbj09	4.9	5.0	4.8	434	41	414	other
sbj09	4.9	4.6	5.2	414	13	343	other
sbj10	4.4	4.0	4.8	430	15	280	other
sbj10	4.2	4.2	4.2	456	34	326	other
sbj10	3.9	3.6	4.2	439	13	316	other
sbj11	4.6	4.6	4.6	374	361	361	M1$_{hand}$
sbj11	5.5	5.0	6.0	302	33	252	other
sbj11	5.6	5.8	5.4	292	11	266	other
sbj12	3.7	3.4	4.0	397	348	348	M1$_{hand}$
sbj12	4.4	4.4	4.4	362	25	268	other
sbj12	4.6	4.8	4.4	367	10	274	other
sbj13	4.4	4.2	4.6	430	395	395	M1$_{hand}$
sbj13	5.3	5.8	4.8	343	23	272	other
sbj13	5.0	5.2	4.8	361	14	280	other
sbj14	5.1	4.6	5.6	396	26	386	other
sbj14	4.3	4.4	4.2	452	449	449	M1$_{hand}$
sbj14	5.0	5.0	5.0	412	18	372	other
sbj16	5.2	4.4	6.0	386	314	314	M1$_{hand}$
sbj16	4.4	3.8	5.0	379	18	362	other
sbj16	3.2	3.4	3.0	386	22	317	other

S13 Table. Simulated pressure values for each trajectory used with ultrasound. *Data for both experiments included. Included are full width half maximum (FWHM) values [mm] of width of the ellipsoid focus of the focused ultrasound beam. The maximum pressures for three key locations for the simulation are also shown: the maximum pressure anywhere, at* $M1_{hand}$*, and at the target coordinate used to aim the trajectory. In some cases, the trajectory coordinate and the* $M1_{hand}$ *coordinate are the same ('Target' column).*
