# Supplementary material for: Ultrasound stimulation of the motor cortex during tonic muscle contraction
Source: PLoS One. 2022 Apr 20;17(4):e0267268. doi: 10.1371/journal.pone.0267268 (PMC9020726; doi:10.1371/journal.pone.0267268)
Supplement: S2 Table — Acoustic properties for media represented in k-Wave. (PDF) [file pone.0267268.s021.pdf]

k-Wave parameters:

Medium properties

	Density [kg/m3]	Speed of Sound[m/s]	Alpha Coefficient [dB/(MHz^y cm)]
**Skull**	1732	2850	8.83
**Brain**	1546.3	1035	0.645946
**Water**	998	1482	6.7403×10^{-5}

Alpha Power (^{y}): 1.43

S16 Table. k-Wave Parameters. *Acoustic properties for media represented in k-Wave.*
