# Supplementary material for: Ultrasound stimulation of the motor cortex during tonic muscle contraction
Source: PLoS One. 2022 Apr 20;17(4):e0267268. doi: 10.1371/journal.pone.0267268 (PMC9020726; doi:10.1371/journal.pone.0267268)
Supplement: S1 Text — A ‘Yes’ to any question prevented inclusion in the study. (PDF) [file pone.0267268.s022.pdf]

Neurological screening questionnaire

- Do you have any active medical, neurological, or psychiatric diagnosis (such as depression, schizophrenia, or bipolar disorder)?
- Do you have a pacemaker, deep brain stimulator, or other implanted electrical device (including intrauterine devices or braces)?
- Do you have, or have you ever had, a significant head injury or neurological disorder (such as a concussion or seizure disorder)?
- Do you have, or have you ever had, any seizures within the past six months?
- Do you have a family history of seizures?
- Do you have, or have you ever had, a history of alcohol or substance dependence?
- Do you have, or have you ever had, any cognitive impairments?
- Do you take any antidepressant medications (such as Prozac, Zoloft, or tricyclic antidepressants)?
- Do you take any antipsychotic medications?
- Do you take any antiviral medications?
- Do you take any amphetamines (such as Adderall)?
- Do you have a history of fainting?
- Do you have a history of migraines?
- Do you have a chronic pain disorder?
- Are you pregnant, or is there a chance you could become pregnant?
- Are you older than 50 years of age?

S14 Text. Full screening questionnaire used for recruitment (neurological health). *A 'Yes' to any question prevented inclusion in the study.*
