# Supplementary material for: Ultrasound stimulation of the motor cortex during tonic muscle contraction
Source: PLoS One. 2022 Apr 20;17(4):e0267268. doi: 10.1371/journal.pone.0267268 (PMC9020726; doi:10.1371/journal.pone.0267268)
Supplement: S1 File — An estimate of a single participant’s cumulative M1hand exposure was made by multiplying the individual peak pressure at the M1hand voxel for all tUS trajectories by the time the tUS device was on for that location. (PDF) [file pone.0267268.s023.pdf]

Exposure formula:

$$\sum_{traj\,=\,1}^{n} P_{traj} \times Time_{traj}$$

$\boldsymbol{n}$: Number of tUS trajectories

$\boldsymbol{P_{traj}}$: Pressure (est.) at $M1_{hand}$ voxel

$\boldsymbol{Time_{traj}}$: tUs-on time for that trajectory

S15 Equation. Exposure formula. *An estimate of a single participant's cumulative* $M1_{hand}$ *exposure was made by multiplying the individual peak pressure at the* $M1_{hand}$ *voxel for all tUS trajectories by the time the tUS device was on for that location.*
