# Supplementary material for: Ultrasound stimulation of the motor cortex during tonic muscle contraction
Source: PLoS One. 2022 Apr 20;17(4):e0267268. doi: 10.1371/journal.pone.0267268 (PMC9020726; doi:10.1371/journal.pone.0267268)
Supplement: Attachment — Submitted filename: PONE-D-21-31823_review.docx [file pone.0267268.s024.docx]

The study investigated the effects of tUS on the primary motor cortex. Specifically, the authors investigated whether tUS could induce cortical silent period similar to the silent period found after TMS during muscle contraction. Parameters of tUS were similar to previous studies, and two sonication durations were tested (300 ms and 500 ms). In a second experiment, using the same parameters as their first experiment, the authors also investigated the effects of ‘offline’ tUS on MEP. This was done by comparing pre-tUS MEPs to post-tUS MEPs. The results showed that tUS did not induce cortical silent periods and there were no ‘offline’ effects of tUS. Finally, the authors conducted simulations that showed that their tUS was effective in targeting M1 hand area.

In spite of the negative results, I do believe that this paper will be a valuable contribution to the literature since our current understanding of tUS on M1 is limited. The experiments were well designed, and the methods are solid. The manuscript is well written, however there are some concerns that needs to be addressed.

**Concerns:**

1. One of the major concerns that I have with the manuscript is the lack of discussion of a paper by Fomenko et al. (2020). Fomenko et al. have systematically investigated the effects of tUS on M1. Importantly they have also tested the effects of tUS on cortical silent period.

Could the author provide a description of how their study expands on those of Fomenko et al. (2020). This should also be included in the discussion section.

As Fomenko et al. (2020) mentions, cortical silent periods are thought to be a marker of GABA_B_ activity. Fomenko et al. (2020) also did not find an effect of tUS on LICI, another marker of GABA_B_ activity. Based on their results Fomenko et al. argues that this suggests that tUS affects the ligand-gated ionotropic GABA_A_ channel rather than GABA_B_. Given this, could the author explain why they would anticipate a different result from Fomenko et al. (2020) or perhaps provide a rationale for their study in relation to the results of Fomenko et al.

Fomenko, A., Chen, K. H. S., Nankoo, J. F., Saravanamuttu, J., Wang, Y., El-Baba, M., ... & Chen, R. (2020). Systematic examination of low-intensity ultrasound parameters on human motor cortex excitability and behavior. *Elife*, *9*, e54497.

2. Page 3 Line 42: The authors claim that the effects of tUS on M1 remains unclear. Could the author provide more details about what they mean by this? Is this specifically referring to overt behaviours or does it include effects on MEP?
3. The tUS parameters are a bit unclear. Specifically referring to the 10s interval (See Page 20 Line 458). Could the author provide a figure that includes this 10 s interval? More details should also be provided for the figure captions.
4. The quality of the figures should be improved. On my version they are quite poor.
5. A major concern that the authors do acknowledge is the small sample size (relative to non-invasive stimulation studies). Could the author explain why they did not include a larger sample size? This is particularly important given the negative results.
