# Supplementary material for: Ultrasound stimulation of the motor cortex during tonic muscle contraction
Source: PLoS One. 2022 Apr 20;17(4):e0267268. doi: 10.1371/journal.pone.0267268 (PMC9020726; doi:10.1371/journal.pone.0267268)
Supplement: Attachment — Submitted filename: ResponsetoReviewers_Revision1.docx [file pone.0267268.s025.docx]

# Response to Reviewers

PONE-D-21-31823

*Ultrasound stimulation of the motor cortex during tonic muscle contraction*

PLOS ONE

December 30, 2021

We would like to thank the reviewers and the editor for their helpful feedback on the manuscript. Your feedback was thorough, including both focused points to improve understanding of specific sections and broader points to touch on questions concerning the field. In particular, we would like to thank you all for bringing *Fomenko, Chen et al., 2020* further into our attention.

We feel that your feedback has allowed us to improve clarity throughout the manuscript. Additionally, it has helped us more fully cover details that tUS researchers may wish to know about our work, as well as strengthening the manuscript’s scientific discussion. For that, we are truly appreciative.

To organize our responses, we took the approach of responding to comments directly, while grouping when appropriate. Following discussion of grouped comments, discussion of individual comments is organized in a table format.

## Sample Size

### Reviewer comments:

*Reviewer 1*

*5. A major concern that the authors do acknowledge is the small sample size (relative to non-invasive stimulation studies). Could the author explain why they did not include a larger sample size? This is particularly important given the negative results.*

*Editor*

*There is significant limitation with the low number of subjects which should be acknowledged or increase the number of subjects.*

### Response:

The small sample size was to meet IRB request to limit sample size and to approach this investigation as a pilot study. The IRB requested this because, in their view, transcranial ultrasound for neuromodulation was a relatively novel technique (especially when we began IRB approval), and our device is not FDA approved. While they approved an amendment for increased enrollment after safety of the first n = 10 was shown, enrollment had to be stopped due to the ongoing coronavirus pandemic.

To compensate for the small sample size, we made use of elaborate methods to account for acoustic penetration variability (i.e. 3D acoustic simulation using subject-specific images), statistically responsible approaches (i.e. non-parametric null hypothesis testing using resampling), and showing data points in figures whenever possible to show that there were no emerging trends.

The editor notes that the limitation with the low number of subjects should be acknowledged. We do note this cumulatively at original lines 509-511:

“While the negative tUS results reported here mirror struggles other investigators have shown when attempting to elicit measurable modulation of M1 by tUS, this was also a pilot study with small sample sizes (n = 8; n = 10). As such, clearer results may emerge with larger datasets or changes in methodology.”

As for acknowledging the sample size at an experiment-by-experiment basis:

Regarding Experiment 1, we demonstrated through multiple means (Fig 2, Fig 3, S2 Fig, S3 Fig) and even showing per subject (S3 Fig) and per trial (S4 Fig) that there is not even a hint of a trend—even among a single subject or trial. It is difficult to conceive what sample size would change the findings of Experiment 1.

Regarding Experiment 2A (Cortical excitability), we did not directly acknowledge the sample size issue by itself (only as a whole on original lines 509-511), so we will note it here. While a larger sample size would have been ideal, within individual subjects we saw a roughly even split of minor increases and decreases of MEP size. Again, it is hard to conceive how a larger sample size would reveal an underlying trend. It is likely that the tUS protocol was not ideal for producing offline effects, which we now more clearly state in the revised manuscript.

Regarding Experiment 2B (Exposure vs. excitability), we note that the study design was a bigger limitation than the sample size. Original lines 474-480:

“With this small sample size (n = 8), we saw no correlation between M1_hand_ exposure and cortical excitability change, though the calculation of this correlation was also low-powered. This conclusion is to be expected since this study was not designed to investigate such a correlation, with M1_hand_ exposure effectively stratified into two levels depending on whether the M1_hand_ target was used once or twice (i.e. whether it was the primary NIBS target, “TMS target”). Studies that wish to investigate potential correlation effects of exposure levels would need to expose participants at a variety of different levels and have a larger sample size than used here to increase statistical power.”

We would also like to note that, though we could not reject the null hypothesis, we do not make a negative statement (i.e. we never say that tUS does not suppress activity).

## Rationale for Experiment 2 parameters

### Reviewer comments:

*Editor:*

*The rationale for Experiment 2 as to why the chosen parameters are expected to produce offline effects should be more clearly stated.*

*Reviewer 2 (From summary):*

*While the justification for Experiment 1 is clear, the justification for the protocol in Experiment 2 (with relatively long inter-trial intervals) is unclear, as there is no indication in the literature that such a protocol with TUS (nor TMS) would result in offline effects that outlast the stimulation."*

### Response:

We have added rationale for Experiment 2 parameters to Methods (Experiment 2, Cortical excitability) and Discussion (Cortical excitability).

Experiment 2 was designed to allow for testing offline effects of *one* protocol without increasing the number of people who would be exposed to tUS. Specifically, Experiment 2 was testing for offline effects produced by the tUS component of Experiment 1, which is why the protocol was the long-interval protocol. We did not expect online effects one way or the other from Experiment 2, as it was not optimized as such. But even though it wasn't optimized, the authors believe it is still valid and interesting data, especially considering how little data there is in humans regarding offline effects with tUS.

## Regarding *Fomenko, Chen et al., 2020*

### Reviewer comments:

*Reviewer 2:
2. Lines 61 to 69 – would be good to cite Fomenko et al. 2020 and Xia et al. 2021 which also looked at TUS effects on M1.*

*Editor:*

*There should be more throughout discussion of relevant published papers (two were pointed out by the reviewers).*

*Reviewer 1:*

*1. One of the major concerns that I have with the manuscript is the lack of discussion of a paper by Fomenko et al. (2020). Fomenko et al. have systematically investigated the effects of tUS on M1. Importantly they have also tested the effects of tUS on cortical silent period.*

*Could the author provide a description of how their study expands on those of Fomenko et al. (2020). This should also be included in the discussion section.*

*As Fomenko et al. (2020) mentions, cortical silent periods are thought to be a marker of GABAB activity. Fomenko et al. (2020) also did not find an effect of tUS on LICI, another marker of GABAB activity. Based on their results Fomenko et al. argues that this suggests that tUS affects the ligand-gated ionotropic GABAA channel rather than GABAB. Given this, could the author explain why they would anticipate a different result from Fomenko et al. (2020) or perhaps provide a rationale for their study in relation to the results of Fomenko et al.*

*Reviewer 2:*

*3. Lines 70 to 77 – it would be helpful to discuss the effects (or lack thereof) of TUS on SICI (Legon et al. 2018 and Fomenko et al. 2020).*

### Response:

Thank you all for the very helpful feedback highlighted here.

We have heavily rewritten the Discussion subsection "Cortical silent period, tUS" to thoroughly discuss *Fomenko, Chen et al., 2020*. We have also added citations for *Fomenko, Chen et al.* and *Xia et al., 2021* throughout the paper, including in the intro as suggested by Reviewer 2.

Regarding the request to "explain why they would anticipate a different result from Fomenko et al. (2020) or perhaps provide a rationale for their study in relation to the results of Fomenko et al.":

For context, the study presented here began before Fomenko, Chen et al. 2020 was published, so we were unable to take its insight into account during study design.

But even now having the insight of *Fomenko, Chen et al.*, the two studies are inherently different, so we have included a discussion of their differences/similarities in the re-written Discussion subsection "Cortical silent period, tUS".

We also added discussion of GABA_A_/GABA_B_, SICI, and our data to the discussion subsection "Cortical silent period, tUS".

## Individual Comments:

Reviewer 1

| **Comment** | **Response to Reviewers** | **Action Taken** |
| --- | --- | --- |
| *2. Page 3 Line 42: The authors claim that the effects of tUS on M1 remains unclear. Could the author provide more details about what they mean by this? Is this specifically referring to overt behaviours or does it include effects on MEP?* | Adjusted the wording of the sentence in question.  Since the sentence in question is at the beginning of the Introduction, we would prefer to not overwhelm the reader with more details. Such details come later in the Introduction and the Discussion. We have softened the wording, however. | Reword |
| *3. The tUS parameters are a bit unclear. Specifically referring to the 10s interval (See Page 20 Line 458). Could the author provide a figure that includes this 10 s interval? More details should also be provided for the figure captions.* | Revised Fig 1 to clarify tUS parameters. This includes the addition of a Panel C that covers the 10-second tUS interval in question. Additional details were also added to the figure description for all sections of Fig 1. | Revised Fig 1 |
| *4. The quality of the figures should be improved. On my version they are quite poor.* | The authors see no quality issues on files downloaded from the editorial manager. Figures were processed through the PLOS One-provided PACE figure assessment tool before uploading. | None |

Reviewer 2

| **Comment** | **Response to Reviewers** | **Action Taken** |
| --- | --- | --- |
| *1. Line 64 – we don’t know whether these intensities are unsafe. They just haven’t been used in humans yet.* | We have removed the sentence in question. | Deleted sentence |
| *4. Lines 81 to 82 – better to state explicitly here already that Experiment 2 is about the after-effects (offline effects) of TUS* | Rephrased sentences in question to state Experiment 2 is about offline effects | Reword |
| *5. In Experiment 1, was the order of TUS vs TMS blocks counterbalanced? Were there any sham/ control trials between TUS or TMS trials?* | No, the order of tUS vs. TMS-cSP blocks were not counter balanced since counterbalancing Experiment 1 blocks would have conflicted with acquisition of Experiment 2.  As for sham for tUS, we recorded EMG for 1000 ms surrounding the 300- or 500-ms tUS burst. We used the tUS-off portions of the 1000-ms epochs as the control. | None |
| *6. Lines 105-115 – It sounds like the motor hotspot search was entirely based on a couple for predefined targets and not really optimized iteratively to find the best coil position and orientation for good, stable, and well-formed MEPs. Is it possible that the optimal TME MEP hotspot was not determined and application of them more focal TUS at those locations was therefore ineffective?*  *Please show the MEP waveform averages for all subjects in the supplement.* | We agree. We discuss this concept at original lines 481-487:  “While we performed acoustic simulation on 907 cm^3^ volumes (~300-350 cm^3^ of which were grey or white matter), we still chose to tabulate cumulative exposure for a single location: M1_hand_. M1_hand_ was chosen because it is the most reasonable small-volume, easily identifiable cortical area that is known to play a direct role in hand muscle contraction. It is, however, possible that directly targeting M1_hand_ may not be the ideal choice for modulating voluntary muscle contraction with tUS. This notion of placement is especially important given the spatially precise nature of tUS compared to a TMS or transcranial electrical stimulation—especially with a small, focused ultrasound transducer as used here.”  Given TMS’s interactions with anatomy, neuron orientation, etc., peak MEP-producing TMS effects do not necessarily occur geometrically on the trajectory the TMS coil is pointing at. As such, we did not want to set our narrow tUS focus based on a TMS-determined hotspot. As such, we chose a scientifically reasonable and identifiable target to aim at: M1_hand_.  Added figure, S17 Fig, of waveform averages and all waveforms for Experiment 2 to Supporting Information. | Added supporting figure |
| *7. Lines 129 to 136 – was the TMS-induced MEP measured before and after an entire block of 20 TUS trials?* | Reworded the sentence in question to improve clarity that yes, "20 MEPs were acquired both before and after the tUS exposure protocol." Also added clarification to the preceeding paragraph and the paragraph in question that there are 80 total tUS trials per person | Reword.  Added clarification |
| *8. Lines 137 to 144 – What specific coupling medium was used (gel, gel pads, water bladder, etc.) and how was good coupling ensured in the presence of hair?* | Added requested details to paragraph in question.  If Reviewer 2 is referring to the technique used in Fomenko, Chen 2021: That technique is smart and good practice, but it is by no means ubiquitous across tUS studies (though perhaps it should be). | Added details |
| *9. Line 139 – is the focus 3 cm long, or 3cm away from the transducer face? Please provide both values, in addition to the width of the focus.* | Added requested details for the focus dimensions. | Added details |
| *10. Line 153 – what attenuation factor is used to estimate in the intracranial intensity? Was it the equation on line 254? Please also provide the Ispta.* | Added requested details.  Reworded to give an exact estimate of Isppa, rather than the ceiling we wanted our estimate to be below (4.9 Isppa from *Legon et al., 2014*). | Added details |
| *11. Lines 169 to 182 – the descriptions of cSP onset and offset determination is difficult to understand. For instance, why would the ‘first rising EMG value’ be used as onset? Please try to use a figure to explain this. And also mention whether similar methods have been used in other papers.* | We rearranged/rewrote the section in question. Specifically, we separated rules regarding edge cases from the main method (falling below a threshold level of EMG activity), which the authors believe has significantly improved clarity.  With the addition of improved wording, the authors believe S1 Fig serves well as a figure to illustrate the method.  We also added mentions of other papers whose methods are similar to ours. | Reword section. |
| *12. Line 189 – why the mean of the two flanking valleys instead of the larger valley? Does this method have any advantages, and has it been previously used?* | Added sentence explaining reason. Follows sentence in question. | Added sentence |
| *13. Line 210 – what is the criteria for determining ‘silence’? Is it different from the threshold used to determine cSP onset and offset?* | Added clarification that yes, silence was calculated "with the same sliding window approach used to determine cSP onset and offset (see cSP measurement)." | Added clarification |
| *14. Please specify the exact MR acquisition parameters for the anatomical MRIs that were used for neuronavigation and acoustic simulation. Please also provide examples of the skull masks overlayed on the subject specific MRIs.* | Added MR parameters to "Skull mask processing" section.  Added supporting figure, S21 Fig, with examples of skull masks overlayed on the subject-specific MRIs | Added details.  Added supporting figure. |
| *15. Line 279 to 282 – what about the TUS trials?* | Added clarification that Original Lines 283-284 pertain to tUS trials. | Added clarification |
| *16. Line 283 – can you please elaborate how ‘spike rate’ is measured? Is this the ‘rate of EMG peaks’ described earlier? Does this measure have any specific physiological significance?* | Yes, it is just the rate of EMG peaks described earlier. Corrected to "rate of EMG peaks" | Added clarification |
| *17. Line 396 – it is likely that some early studies recorded responses to the acoustic rather than neuromodulatory effects of TUS. However, recent papers have confirmed that neuromodulation is also present (see for eg. Mohammadjavadi et al. 2019, Brain Stimulation). I recommend adding some nuance to this statement.*  *On the topic of sensory confounds, did any participants in your study report auditory and/ or somatosensory perception accompanying the TUS trials? Have they been asked explicitly?* | Added nuance and reference to *Mohammadjavadi et al. 2019* at section in question.  We did not ask all participants, but the authors do not believe this is of concern since our study did not concern subject performance or self-reported outcomes. Subjects we did ask did not notice anything.  What subjects *did* mention was the task (finger abduction at a constant force) was somewhat difficult. As such, subjects were very focused on maintaining the constant force output during tUS trials, so we doubt they would have had the mental overhead to attend to any minor differences in tactile-level vibration.  The fan noise from our amplifier was also quite loud (constant noise level throughout tUS session), which may have served to mask any audible tUS-based vibrations to the ear. | Added nuance.  Added citation. |
| *18. Lines 408 to 409 – again, I recommend adding some nuance to this statement. While TUS may preferentially stimulate inhibitory neurons at some parameter combinations, there is also evidence that it can stimulate excitatory neurons (see Yu et al. 2021, Nature Communications).* | Added nuance and reference to *Yu et al., 2021* in the paragraph in question. | Added nuance.  Added citation. |
| *19. Fig. S3 – it seems like this figure shows fewer peaks in the TMS compared to TUS trials. I presume the amplitude of the peaks where higher in TMS?* | Added clarification to S3 figure description | Added clarification |
| *20. Fig. 5 – Why are MEP AUCs shown on the y-axis as if they only had a very limited number of discrete values? Please alternatively also show (and used for statistics) the more well-established MEP peak-to-peak amplitudes.* | Added supporting figure, S19 Fig, with MEP amplitudes. Added amplitude statistics to Supporting Information (S19 Fig).  Original data are not discrete values. MEPs are binned into discrete vertical-axis bins to allow visualization of the distribution of the data akin to a violin plot. This manuscript takes the approach of showing data points whenever possible, hence why we did not use a violin plot or box plot here. Alternatively a jitter plot or strip plot could have been used.  Added additional description of the binned visualization to Fig 5 description. | Added supporting figure.  Added clarification. |
| *21. Fig. 7. – from the caption it does not become clear what the difference between (a) and (b) is supposed to be. In (a), both TUS beams seem to have “hit” the central sulcus in the hand knob, if anything more post-centrally, whereas in (b) it looks like the focal spot is very anterior and rather in the premotor cortex. None of the presented foci seem to be particularly well targeting the anterior wall of the central sulcus where M1 corticospinal output neurons are located. Simulated acoustic pressure maps for all targets and should be presented for all subjects in the Supplement.* | A very crucial "not" was missing in (b) of the original submission. Thank you for spotting that rather problematic typo. We have fixed the typo.  We also added additional information to the figure.  Added pressure maps of all subjects, all targets as *S18 Images*. | Added supporting figure |
| *22. Fig. 7 – it looks like the skull image for the subj-01 is not complete. Maybe the FOV was cut-off? This has major implications for the quality of the simulations. Please discuss these limitations.* | To produce accurate acoustic simulations with k-Wave, one wants to use as high a resolution as possible. If it’s not high enough, simulation quality will be very bad. As such, we increased resolution through interpolation from 0.8 mm to 0.2 mm. To have accurate simulations, we also performed simulations in 3D. These two things together significantly increase memory load. To compensate, we reduced the FOV to include just the transducer and the focal area. This allows for high-resolution, accurate images at the expense of not simulating areas with very low max pressures (outside the focal area). But as can be seen in Fig 7, the ultrasound energies have mostly been absorbed or dispersed (due to the focused nature of our transducer) by the time they reach the end of the FOV. Beyond the focal point, energies are too low to cause physiological effects detectable in this study. And since this is a focused ultrasound transducer with a short (3 cm) focal length, there is no concern of standing waves bouncing off the back of the skull.  Reviewer 2 specifically mentions sbj01, and not sbj11 as well, as having an issue. As such, the authors will respond to this comment with the assumption that Reviewer 2 is referring to a visually distinguishable difference between the two examples. Specifically, we assume Reviewer 2 is referring to the apparent crop visible at the skull to the far left of the image on the leftmost slices shown. Since we have a small, 3-cm diameter transducer, the cropped area in question is far from the location of the transducer. As such, no pressures interact with the cropped portion of the skull. To demonstrate this, we have added a new file, *S20 Images*, to the Supporting Information. | Added supporting figure |
| *23. Some information seem to be missing in the data availability statement: “All EMG files are available from the XXX database (url: XXX). Code to run acoustic simulations is available via the MATLAB package TUSX (*[*www.tusx.org*](http://www.tusx.org/)*).”* | Data uploaded to OSF ([OSF.io](http://osf.io/)). URL:<https://osf.io/w547y/?view_only=b0a496d7a4464a728cb189f5cd452abd> | Data uploaded |
