# Supplementary material for: Ultrasound stimulation of the motor cortex during tonic muscle contraction
Source: PLoS One. 2022 Apr 20;17(4):e0267268. doi: 10.1371/journal.pone.0267268 (PMC9020726; doi:10.1371/journal.pone.0267268)
Supplement: Attachment — Submitted filename: Round2_PONE-D-21-31823_review.docx [file pone.0267268.s026.docx]

I commend the authors for taking on my previous comments and for including a discussion of Fomenko et al. (2020) with respect to their results. The paper is almost ready for publication, however, I two major concerns that need to be addressed.

**Major concern:**

Comment 1: While the authors show the simulation results, the study appear to be lacking a major control. Given that we know that tUS reduces MEP at rest, the authors should provide evidence that their tUS was also able to replicate this. Because otherwise, we cannot be sure that the negative results are due to an issue with the equipment (or any other confounds) or because tUS truly does not affect cortical silent period and active MEPs. If the authors do not agree that such a control is necessary, could they explain their reasoning, and include a couple of sentences in the discussion.

**Minor concerns:**

Comment 1: While the study discusses the results of Gibson et al. with respect to offline effects, a recent paper by Zeng et al. (2022) should also be included in this discussion.

Zeng, K., Darmani, G., Fomenko, A., Xia, X., Tran, S., Nankoo, J. F., ... & Chen, R. (2022). Induction of human motor cortex plasticity by theta burst transcranial ultrasound stimulation. *Annals of neurology*, *91*(2), 238-252.

Comment 2: Could the author provide a schematic of the experimental design of Experiment 2. It is hard to follow as it is written.

Comment 3: What was the coupling medium used to apply tUS?

Comment 4: Was there any auditory mask used? A sentence or two about this is important.

Comment 5: I just want to commend the authors for making the Matlab TUSX toolbox. I am sure it will be very useful to researchers.
