# Supplementary material for: Ultrasound stimulation of the motor cortex during tonic muscle contraction
Source: PLoS One. 2022 Apr 20;17(4):e0267268. doi: 10.1371/journal.pone.0267268 (PMC9020726; doi:10.1371/journal.pone.0267268)
Supplement: Attachment — Submitted filename: ResponsetoReviewers_Revision2.docx [file pone.0267268.s027.docx]

# Response to Reviewers

PONE-D-21-31823

*Ultrasound stimulation of the motor cortex during tonic muscle contraction*

PLOS ONE

March 25, 2022

We would like to thank the reviewers and the editor for their helpful feedback on the revised manuscript. We have taken action on all comments given.

Discussion of comments is organized in a table format.

## Individual Comments:

Reviewer 1

| **Comment** | **Response to Reviewers** | **Action Taken** |
| --- | --- | --- |
| *Major concern:  Comment 1: While the authors show the simulation results, the study appear to be lacking a major control. Given that we know that tUS reduces MEP at rest, the authors should provide evidence that their tUS was also able to replicate this. Because otherwise, we cannot be sure that the negative results are due to an issue with the equipment (or any other confounds) or because tUS truly does not affect cortical silent period and active MEPs. If the authors do not agree that such a control is necessary, could they explain their reasoning, and include a couple of sentences in the discussion.* | We would be happy to attempt to replicate the findings recently published paper (*Zeng et al., 2022*), but unfortunately, the lead author of this paper, Dr. Ian Heimbuch, has joined a new lab for his postdoc. His departure makes the execution of the suggested control experiment rather difficult, from a practical standpoint.  Furthermore, our study is really about the potential effect of tUS in inducing the cortical silent period. While it is exciting that another group has observed that tUS reduces resting MEPs with a tUS protocol quite different from ours, this observation is somewhat tangential to our experimental design. However, since it is relevant to the general issue of the role of tUS in modulating the motor system, we now mention the new study in our revised manuscript. | Added to discussion |
| *Minor concerns:  Comment 1: While the study discusses the results of Gibson et al. with respect to offline effects, a recent paper by Zeng et al. (2022) should also be included in this discussion.  Zeng, K., Darmani, G., Fomenko, A., Xia, X., Tran, S., Nankoo, J. F., ... & Chen, R. (2022). Induction of human motor cortex plasticity by theta burst transcranial ultrasound stimulation. Annals of neurology, 91(2), 238-252.* | Added discussion of the Feb 2022 paper *Zeng et al.* to the Discussion section (Revision 2, Lines 570-574). Citations to *Zeng et al.* have been added to the Discussion and Conclusions section. | Added to discussion |
| *Comment 2: Could the author provide a schematic of the experimental design of Experiment 2. It is hard to follow as it is written.* | We have added a supporting figure (S23 Fig) that demonstrates the sequence of Experiments 1 and 2. We have also reworded the most problematic sentences (Rev. 1 Lines 152-156).  We also added a separate supporting figure (S22 Fig), which may also aid readers who wish to have further clarification. | Added two supporting figures |
| *Comment 3: What was the coupling medium used to apply tUS?* | As stated on Revision 1 line 167, “ultrasound gel was used to acoustically couple the transducer to the subject’s head. | No change |
| *Comment 4: Was there any auditory mask used? A sentence or two about this is important.* | Added two sentences within the Methods section that describe how our very loud rack-mounted amplifier produced fan noise at a constant level, thus acting as an auditory mask.  Considering we show only null results for tUS, we believe this auditory mask is sufficient. | Added two sentences to Methods |
| *Comment 5: I just want to commend the authors for making the Matlab TUSX toolbox. I am sure it will be very useful to researchers.* | Thank you |  |

Reviewer 2

| **Comment** | **Response to Reviewers** | **Action Taken** |
| --- | --- | --- |
| *Reviewer #2: Tha authors have adequately addressed most of my concerns. I have only some minor comments remaining:*  *1. The heading exposure vs. excitability is confusing. A suggestion is to say ‘association between exposure and excitability’ instead.* | Good point. We changed the headings in question to "Exposure & excitability". | Renamed heading |
| *2. Can you please provide some supporting evidence for this statement –*  *‘In a scenario of on ongoing voluntary motor activity, a subset of interneurons in M1 will have already been recruited, so perhaps the channels or cells that tUS would have affected are already being actively modulated by the time tUS energies into the tissue (e.g. channels have already been opened).’*  *For instance, is there evidence from TMS studies suggesting that inhibitory interneurons are already active during a voluntary muscle contraction?* | Thank you for asking to clarify this point. There is evidence from optogenetic tagging that interneurons increase activity during voluntary motor behavior (reaching) (*Estebanez et al. 2017, Cell Reports*). We revised the manuscript to clarify this issue. | Adjusted wording.  Added sources. |
| *3. Please include the information about counter-balancing and sham in the manuscript.* | Added requested info to Methods. Counterbalancing at Revision 2, Line 144. tUS control at Revision 2, Line 331. | Added two sentences to Methods |
